# Characteristics of T-Cells Expressing IL-37 and Its Receptors in Inflammatory Bowel Disease

**DOI:** 10.3390/ijms27031540

**Published:** 2026-02-04

**Authors:** Indiana Zorkau, Peter J. Eggenhuizen, Marie Lee, Steven X. Cho, Kylie R. James, Andrew M. Ellisdon, James C. Whisstock, Joshua D. Ooi, Marcel F. Nold, Claudia A. Nold-Petry, Rimma Goldberg

**Affiliations:** 1 Centre for Inflammatory Diseases, Department of Medicine, School of Clinical Sciences, Monash University, Clayton, VIC 3168, Australia; 2 The Ritchie Centre, Hudson Institute of Medical Research, Clayton, VIC 3168, Australia; 3 Department of Paediatrics, Monash University, Clayton, VIC 3168, Australia; 4 Garvan Institute of Medical Research, Darlinghurst, NSW 2010, Australia; 5 School of Biomedical Sciences, University of New South Wales, Sydney, NSW 2052, Australia; 6 Biomedicine Discovery Institute, Monash University, Clayton, VIC 3800, Australia; 7 Monash Children’s Hospital, Clayton, VIC 3168, Australia; 8 Department of Gastroenterology, Monash Health, Clayton, VIC 3168, Australia

**Keywords:** IBD, IL-37, T-cell

## Abstract

IBD pathogenesis is underpinned by an imbalance between excess inflammation caused by effector T-cells and inadequate suppression by regulatory T-cells (Tregs). Interleukin-37 (IL-37) is a potent, anti-inflammatory cytokine that signals via its receptors IL-1R5 and IL-1R8. Hence, augmenting anti-inflammatory mechanisms that drive IL-37 expression is a strategy to control IBD-associated inflammation. However, the role of IL-37 and its receptors in T-cells remains incompletely understood. Here, we investigated T-cell expression profiles of IL-37 and its receptors to understand the drivers of dysregulated T-cell responses in IBD and develop novel, more effective therapies. T-cell subsets from healthy control (HC), Crohn’s disease (CD) and ulcerative colitis (UC) peripheral blood mononuclear cells (PBMC) and lamina propria mononuclear cells (LPMC) were assessed for expression of IL-37 and its receptors by flow cytometry. CD3+IL-1R8+ T-cell transcriptomes underwent RNA sequencing. The phenotype and suppressive capacity of Tregs supplemented with IL-37 was assessed in vitro. Our results indicate that IL-37 and its receptors were differentially expressed among PBMC and LPMC T-cell subsets in IBD patients compared to HC. Transcription signatures unique to IBD were revealed, particularly histone and mitochondrial pathways. Remarkably, culturing Tregs with IL-37 preserved FOXP3 expression and suppressiveness at a level comparable to treatment with the well-established Treg stabilizing agent rapamycin. Altogether, our study identified differences in T-cells expressing IL-37 and its receptors that are indicative of T-cell dysfunction in IBD. These findings highlight a novel and promising avenue for restoring immune homeostasis in IBD by targeting and boosting the IL-37 signalling pathway.

## 1. Introduction

Inflammatory bowel disease (IBD) encompasses two main chronic autoimmune diseases of the gastrointestinal tract, Crohn’s disease (CD) and ulcerative colitis (UC), both of which lack a cure. IBD affects roughly 0.3% of the Western population [1], with patients experiencing disease flares interspersed with periods of remission. Current therapies for IBD include 5-aminosalicylic acid, corticosteroids, immunomodulators and biologics such as anti-tumour necrosis factor (e.g., infliximab), anti-α4β7 integrin (e.g., vedolizumab) and anti-interleukins (e.g., ustekinumab, blocking IL-12 and IL-23) [2]. However, not all patients are responsive to current therapies, with a third not responding to first-line treatment and half of the non-responders losing response to second-line treatments [3]. These high rates of treatment failure contribute to the challenge IBD patients face in achieving stable disease management. Consequently, there is a need for novel therapies, particularly treatments that target the underlying source of immune dysregulation in IBD, especially in terms of anti-inflammatory mediators.

In addition, IBD-related microbial dysbiosis contributes to epithelial damage, abnormal mucus production and defective barrier repair [4,5]. It is postulated that IBD is caused by an inappropriate response to non-pathogenic bacteria in genetically susceptible individuals [6]. Increased exposure to bacterial antigens generally promotes the expansion of pro-inflammatory effector CD4+ T-cells (Teffs) including T helper (Th) 1 and Th17 in CD [7,8,9] and Th2 in UC [10]. The combination of excessive immune infiltration and the loss of anti-inflammatory regulatory mechanisms such as T regulatory cells (Tregs) then drives chronic inflammation in IBD [11,12].

Tregs are a subset of CD4+ T-cells characterised by the transcription factor FOXP3 [13,14]. Tregs maintain immune homeostasis, suppress inflammation through the production of anti-inflammatory cytokines such as interleukin (IL)-10, transforming growth factor (TGF)-β, IL-35 and IL-37, and facilitate Teff suppression through a variety of mechanisms, including inhibiting T-cell activation via CTLA-4, IL-2 deprivation through high expression of the IL-2 receptor CD25, and direct cytolysis by the expression of granzymes and perforin [15]. Peripherally induced Tregs are enriched within the lamina propria and gut-associated lymphoid tissue, where they play a critical role in maintaining immune tolerance to bacterial antigens [16]. In the context of IBD, Tregs act in a dysregulated fashion, inadequately controlling pro-inflammatory responses [11,17]. IBD patients exhibit fewer Tregs [18] and increased Th17 cells in circulation [19]. Previous studies have reported an increase in peripheral Tregs in the mucosal tissue of IBD patients, yet these cells exhibit decreased suppressor functions [20,21]. In addition, Tregs undergo apoptosis more frequently in the LP of IBD patients [22]. We have previously demonstrated that in CD, there is an increase in Teffs trafficking to the gut compared to Tregs [23] and these intestinal Teffs are resistant to suppression by Tregs [24].

IL-37, previously known as IL-1F7, is an anti-inflammatory member of the IL-1 family [25] and inhibits both innate and adaptive immunity [26,27,28]. IL-37 has five isoforms (IL-37a-e), of which IL-37b is the most abundant [25]. Despite being widely regarded as an anti-inflammatory molecule, one modified form of IL-37b has been reported to be pro-inflammatory by signalling via the IL-36 receptor [29]. IL-37 is a dual function cytokine with intracellular functions in addition to receptor-mediating signalling. It can act as an alarmin, being rapidly released upon inflammatory encounters, and also translocates into the nucleus [30,31]. Peripheral blood mononuclear cells (PBMC) have been identified as a source of endogenous IL-37 [32], with monocytes being the most abundant source of IL-37, followed by T-cells [33]. When released from cells, IL-37 binds to its cell surface receptor complex, which is made up of IL-1 receptor 5 (IL-1R5), also known as IL-18 receptor alpha (IL-18Rα), and IL-1 receptor 8 (IL-1R8), also known as single immunoglobulin IL-1-related receptor (SIGIRR) [34]. Multiple studies have determined that IL-37 is expressed in human CD4+CD25+ Tregs [35,36], and downregulation of IL-37 leads to a decrease in FOXP3 expression levels in Tregs [35]. Furthermore, it has been found that recombinant IL-37 leads to enhanced suppressive capabilities of CD4+CD25+ Tregs in mice [37]. As a murine homolog for IL-37 is yet to be discovered, in vivo studies using human IL-37 transgenic mice have shown that IL-37-producing cells increased protection against colitis that was dependent on a balanced microbiome and protected from necrotizing enterocolitis, a severe inflammatory disease of the intestine affecting preterm infants [38,39,40,41]. In the context of human IBD, patients exhibited decreased serum IL-37 during active disease, indicating a reduced ability to control inflammation [42]. Higher levels of inflammation in the colon were shown to increase levels of IL-37 protein expression in human immune and epithelial cells and in human IL-37 transgenic mice, and IL-37 acts in an anti-inflammatory manner on the lamina propria immune cells to protect intestinal barrier integrity [42,43,44,45]. These results indicate that in disease situations such as IBD, the immune system attempts to compensate with anti-inflammatory mechanisms through expression of IL-37. However, when the anti-inflammatory signal is not strong enough or dysregulated, disease ensues. Hence, augmenting the anti-inflammatory mechanisms, such as by enhancing IL-37, is a promising therapeutic strategy to control IBD-associated inflammation. However, current research regarding the role of IL-37 and the effect on different T-cell types, including Tregs, in IBD is limited.

In this study, we aimed to understand the role and abundance of IL-37 and its receptors IL-1R5 and IL-1R8 across T-cell subsets in IBD patients compared to healthy controls (HC). We explored whether gene expression profiles of peripheral blood T-cells expressing IL-1R8 could serve as markers of IBD. In addition, we assessed whether recombinant IL-37 could be used as a biologic therapy to increase Treg-suppressive capacity by comparing the ability of IL-37-treated Tregs to suppress T effector activity in vitro.

## 2. Results

To determine the expression of IL-37 in particular T-cell subtypes, blood and biopsy samples from healthy controls (HCs), CDs and UCs were obtained (*n* = 18, 15 and 14, respectively), with patient demographics outlined in Table 1.

### 2.1. IL-37 and Its Receptors Are Differentially Expressed Among Peripheral Blood T-Cell Subsets in IBD

#### 2.1.1. IL-37

**Figure 1:**
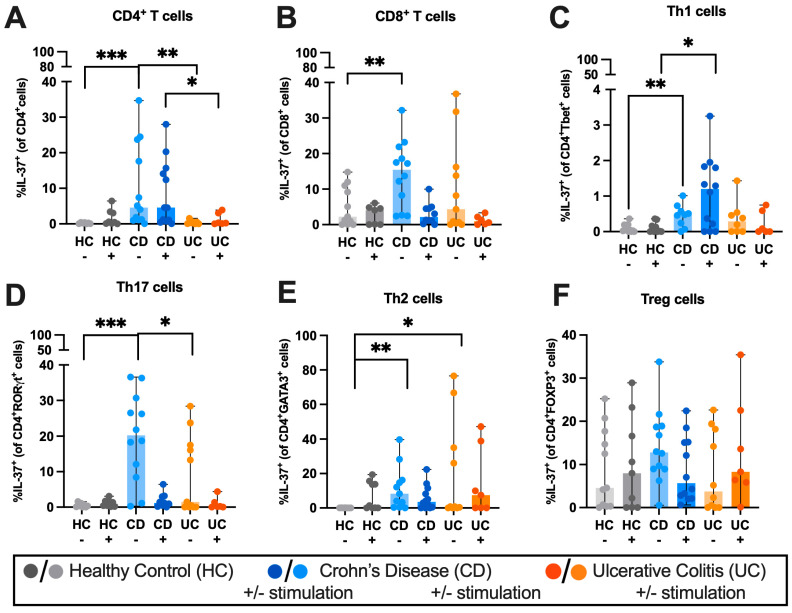
IL-37 is differentially expressed in PBMC-derived T-cell subsets in IBD. PBMCs were either unstimulated or stimulated with PMA (50 ng/mL) and Ionomycin (750 ng/mL), with the addition of brefeldin A (10 μg/mL), for 4 h. Percentage of IL-37 expression in PBMC-derived (**A**) total CD4+ T-cells, (**B**) CD8+ T-cells, (**C**) Th1 cells (of CD4+ Tbet+ T-cells), (**D**) Th17 cells (of CD4+ RORγt+ T-cells), (**E**) Th2 cells (of CD4+ GATA3+ T-cells) and (**F**) Treg cells (of CD4+ FOXP3+ T-cells). Mann-Whitney U tests were used to determine statistical significance among patient groups. Healthy control (HC) *n* = 12, Crohn’s disease (CD) *n* = 12 and ulcerative colitis (UC) *n* = 11. Outliers were removed using the ROUT outliers test. Data is represented as median ± range. * *p* < 0.05, ** *p* < 0.005 and *** *p* < 0.0005.

To determine the proportion of T-cell subsets expressing IL-37, IL-1R5 and IL-1R8 in IBD, we performed flow cytometry on stimulated and unstimulated peripheral blood T-cells (see gating strategy in Supplementary Figure S1), which showed that mean IL-37 expression was increased 19.8-fold (*p* = 0.0005) in unstimulated CD4+ T-cells and increased 6.0-fold (*p* = 0.0044) in unstimulated CD8+ T-cells in CD patients compared to HC but not in UC patients (Figure 1A,B). To obtain a better understanding of which sub-type of CD4+ T-cells expresses IL-37, we analysed IL-37 expression in Th1, Th2, Th17 and Treg cells, using their defining transcription factors Tbet for Th1 cells, GATA3 for Th2 cells, RORγt for Th17 cells and FOXP3 for Tregs (Figure 1C–F, Supplementary Figure S2). We found that IL-37 expression in PBMC-derived CD4+ T-cells was most markedly upregulated in unstimulated Th17 cells in CD with a 57.7-fold increase (*p* = 0.0005) compared to HC and unstimulated Th17 cells in UC also showed a 3.3-fold increase (*p* = 0.03) in IL-37 expression. Th1 cells expressed less IL-37 than Th17 but still exhibited a 9.9-fold increase (*p* = 0.0058) in CDs compared to HCs, whereas in Th2 cells, IL-37 was almost undetected in HC samples but was present in both UC (0.79% [0–76.5] vs. 0 [0–0.16], *p* = 0.0472) and CD (8.26% [0–39.7] vs. 0% [0–0.16], *p* = 0.0011) unstimulated cells.

**Figure 2:**
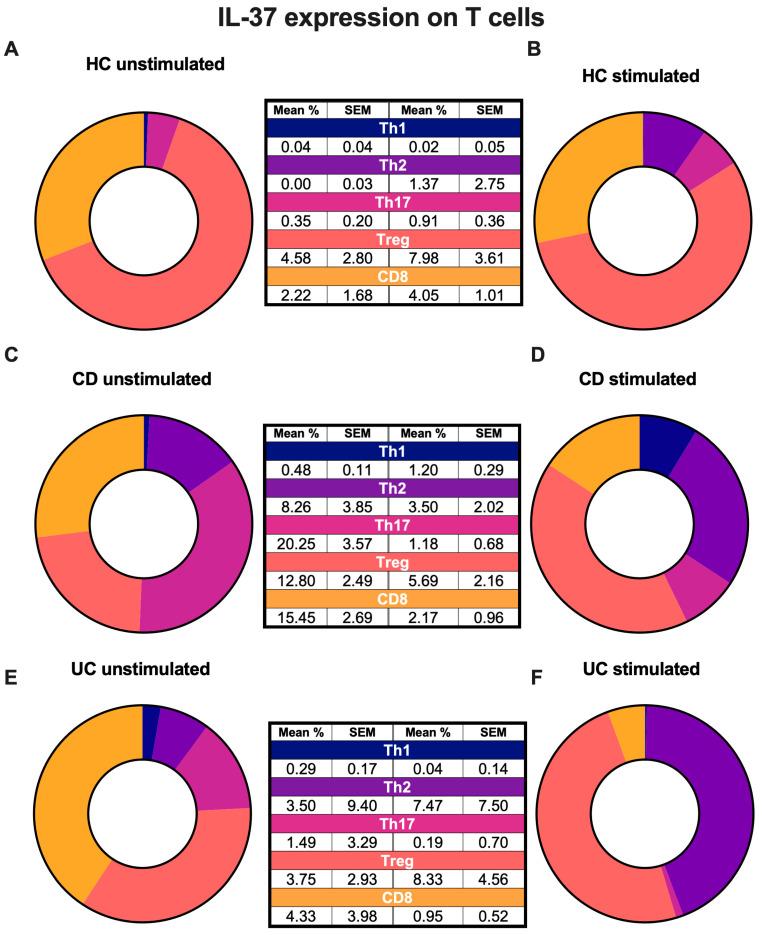
IL-37 expression among peripheral blood T-cell subsets in each disease state. PBMCs were either left untreated or stimulated with PMA (50 ng/mL) and Ionomycin (750 ng/mL), with the addition of brefeldin A (10 μg/mL) for 4 h. Expression of IL-37 shown as mean % and standard error mean (SEM). Donut charts show mean % IL-37+ cells of T-cell subsets in (**A**) HC unstimulated cells, (**B**) HC stimulated cells, (**C**) CD unstimulated cells, (**D**) CD stimulated cells, (**E**) UC unstimulated cells and (**F**) UC stimulated cells. *n* = 12 HC, *n* = 12 CD and *n* = 11 UC. Outliers were removed using the ROUT outliers test.

We then compared the expression of IL-37 from each T-cell subset in each disease state to understand which T-cells expressed IL-37 (Figure 2, Supplementary Figure S3). In unstimulated PBMCs from HCs, the predominant IL-37+ subtype was Treg cells with 4.6% of Tregs being IL-37+ (Figure 2A). However, in unstimulated PBMCs from IBD patients, the pro-inflammatory T-cell subsets Th1, Th2, Th17 and CD8+ T-cells exhibited the highest percentage of IL-37 positivity (Figure 2C,E).

Treg cells expressed significantly more IL-37 than Th1 cells across healthy and IBD groups whether stimulated or not (Supplementary Figure S3). However, the differences were most marked in the unstimulated CD group, with Tregs expressing 25.7-fold more IL-37 than Th1 cells (*p* ≤ 0.0001) (Supplementary Figure S3).

#### 2.1.2. IL-1R5 and IL-1R8

**Figure 3:**
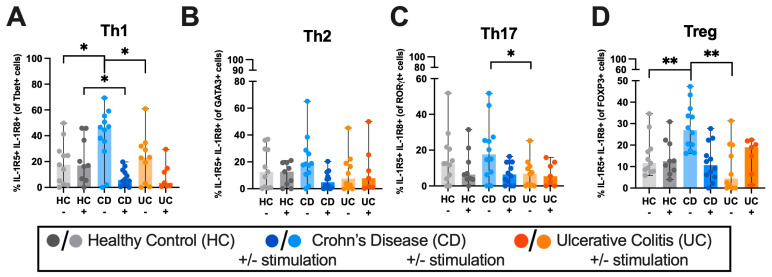
Co-expression of IL-1R5 and IL-1R8 is increased in PBMC Th1, Th17 and Treg cells of CD patients. PBMCs were either left unstimulated or stimulated with PMA (50 ng/mL) and Ionomycin (750 ng/mL), with the addition of brefeldin A (10 μg/mL) for 4 h. Percentage of co-expression of IL-1R8 and IL-1R5 expression in (**A**) Th1 cells, (**B**) Th2 cells, (**C**) Th17 cells and (**D**) Treg cells. Mann-Whitney U tests were used to determine statistical significance among patient groups. *n* = 12 HC, *n* = 12 CD, *n* = 11 UC. Outliers were removed using the ROUT outliers test. Data is represented as median ± range. * *p* < 0.05, ** *p* < 0.005.

The co-expression of the IL-37 receptor, IL-1R5 and IL-1R8 on unstimulated Th1 cells showed a 1.6-fold increase (*p* = 0.023) in CD patients compared to HC and a 1.3-fold increase (*p* = 0.0045) on unstimulated Tregs in CD patients compared to HC (Figure 3A,D). Comparing CD and UC groups, CD patients exhibited increased IL-37 receptor co-expression compared to UC patients, particularly from Treg (5.0-fold increase, *p* = 0.0015), Th17 (1.6-fold increase, *p* = 0.0485) and Th1 (0.98-fold increase, *p* = 0.0437) cells (Figure 3A,C,D). No significant differences in IL-37 receptor co-expression were detected between patient groups on Th2 cells (Figure 3B).

**Figure 4:**
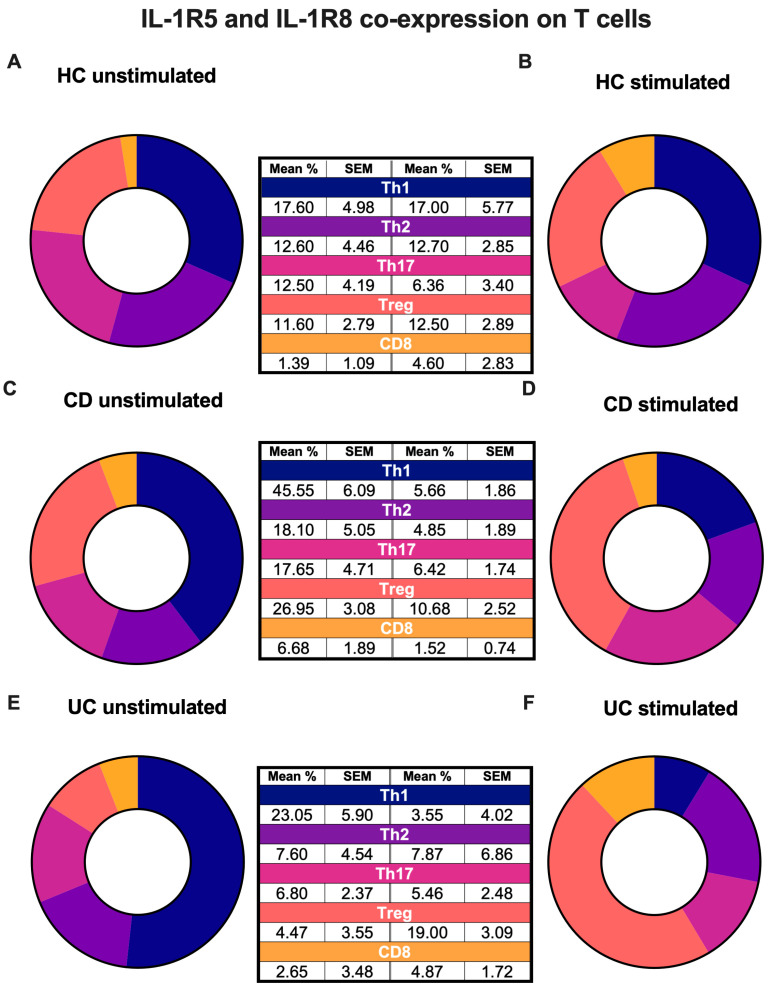
Co-expression of IL-1R5 and IL-1R8 on peripheral blood T-cell subsets in each disease state. PBMCs were either left untreated or stimulated with PMA (50 ng/mL) and Ionomycin (750 ng/mL), with the addition of brefeldin A (10 μg/mL) for 4 h. Co-expression of IL-1R8 and IL-1R5 shown as mean % and standard error mean (SEM). Donut charts show mean % IL-1R8+IL-1R5+ cells of T-cell subsets in (**A**) HC unstimulated cells, (**B**) HC stimulated cells, (**C**) CD unstimulated cells, (**D**) CD stimulated cells, (**E**) UC unstimulated cells and (**F**) UC stimulated cells. *n* = 12 HC, *n* = 12 CD, *n* = 11 UC. Outliers were removed using the ROUT outliers test.

We next compared the co-expression of IL-1R5 and IL-1R8 from each T-cell subset in each disease state to understand which T-cells express the most (Figure 4, Supplementary Figure S4). The co-expression of IL-1R5 and IL-1R8 was higher in unstimulated PBMCs compared to stimulated PBMCs across healthy and IBD patients (Supplementary Figure S4). The co-expression of IL-1R5 and IL-1R8 in all disease states was increased in Treg compared to CD8+ T-cells, with unstimulated Tregs exhibiting mean 7.3-fold (HC, *p* = 0.0002) and 2.9-fold (CD, *p* = 0.0004) greater co-expression of IL-1R5 and IL-1R8 than CD8+ T-cells (Supplementary Figure S4).

### 2.2. LPMC-Derived IL-37 Is Elevated in IBD CD8+ T-Cells, While Its Receptors Are Decreased in IBD CD4+ T-Cells

**Figure 5:**
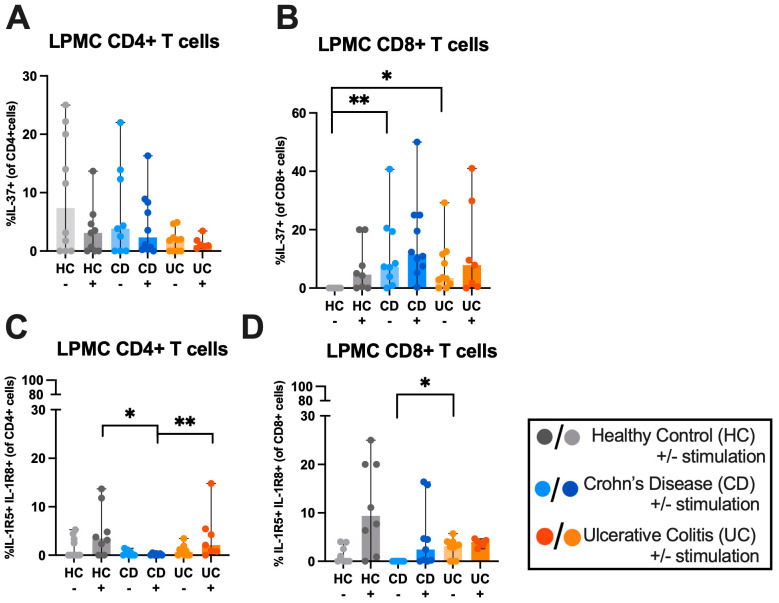
Expression profiling of IL-37 and its receptors in T-cell IBD patient lamina propria mononuclear cells (LPMCs). LPMCs were either left unstimulated or stimulated with PMA (50 ng/mL) and Ionomycin (750 ng/mL), with the addition of brefeldin A (10 μg/mL) for 4 h. LPMC expression of (**A**) IL-37 on CD4+ T-cells, (**B**) IL-37 on CD8+ T-cells, (**C**) co-expression of IL-1R5 and IL-1R8 on CD4+ T-cells and (**D**) co-expression of IL-1R5 and IL-1R8 on CD8+ T-cells. Mann-Whitney U tests were used to determine statistical significance among patient groups. *n* = 12 HC, *n* = 12 CD, *n* = 11 UC. Outliers were removed using the ROUT outliers test. Data is represented as median ± range. * *p* < 0.05, ** *p* < 0.005.

In order to assess the expression of IL-37 and its receptors in gut-derived T-cells, we analysed IL-37 and its receptors IL-1R5 and IL-1R8 in LPMCs from HC and IBD patient (CD and UC) CD4+ and CD8+ T-cells (Figure 5). There was no difference in IL-37 expression on LPMC CD4+ T-cells between CD, UC and HC groups. CD8+ T-cells from LPMC HCs did not express any IL-37, whereas IBD patient unstimulated CD8+ T-cells did express IL-37 with a mean 7.35% increase in CD8+ T-cells compared to CD patients (*p* = 0.0050) and 3.44% increase compared to UC patients (*p* = 0.0123) (Figure 5A,B).

Co-expression of the IL-37 receptors IL-1R5 and IL-1R8 was almost not found in stimulated LPMC-derived CD4+ T-cells of CD patients and was significantly decreased in CD patients compared to both HC (0.00% [0–0.5] vs. 3.44% [0–13.7], *p* = 0.0298) and UC (0.00% [0–0.5] vs. 2.10% [0–14.8], *p* = 0.0099) patients. Co-expression was decreased in unstimulated LPMC CD8+ T-cells from CD patients compared to UC patients (0.00% [0–0] vs. 3.08% [0–5.71], *p* = 0.0181) (Figure 5C,D).

Overall, immunophenotyping of IL-37 and its receptors on PBMC- and LPMC-derived T-cells revealed differential expression in IBD compared to HCs that can be stratified into unique signatures from CD and UC patients.

### 2.3. RNA Sequencing Reveals Transcription Signatures Unique to IBD

**Figure 6:**
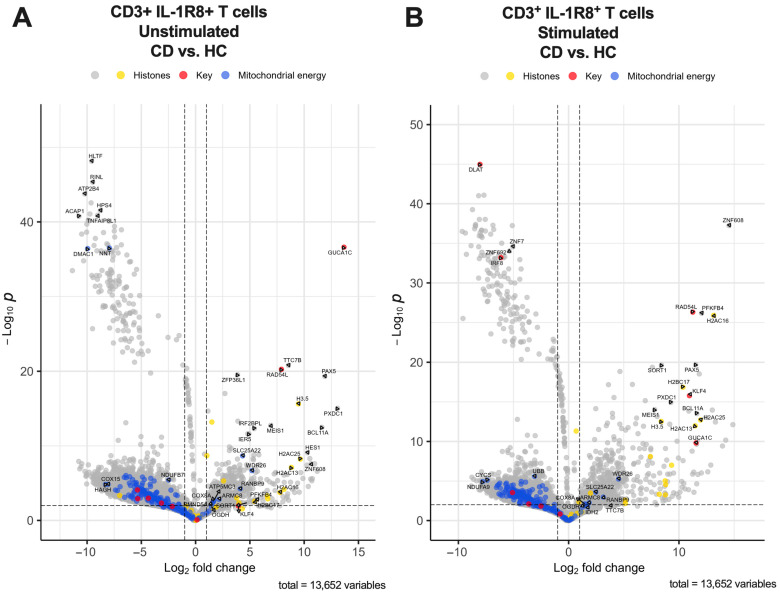
CD3+ IL-1R8+ T-cells from CD PBMCs reveal transcription signatures unique to IBD. Differential gene expression of CD3+ T-cells expressing IL-1R8 from CD patient PBMCs (*n* = 3) reveals key up- and down-regulated genes compared to HCs (*n* = 3, age- and sex-matched). Dotted lines represent the threshold for magnitude of change (X-axis) and significance (Y-axis). (**A**) Volcano plot of differentially expressed genes (DEGs) in unstimulated CD3+IL-1R8+ T-cells in CD samples compared to HC. (**B**) Volcano plot of DEGs in PMA and ionomycin-stimulated CD3+IL-1R8+ T-cells in CD samples compared to HC. Yellow-coloured DEGs are genes related to histone pathways. Blue-coloured DEGs are genes related to mitochondrial energy. Red-coloured DEGs are genes mentioned in the results and discussion. Statistics used are described in Methods under RNAseq analysis.

**Figure 7:**
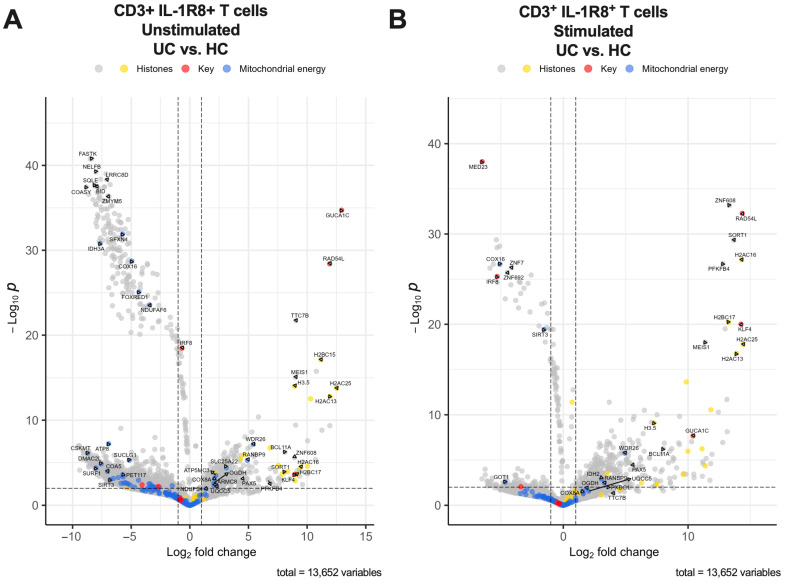
CD3+ IL-1R8+ T-cells from UC PBMCs reveal unique transcription signatures. Differential gene expression of CD3+ T-cells expressing IL-1R8 from UC patient PBMCs (*n* = 3) revealed key up- and down-regulated genes compared to HCs (*n* = 3 age- and sex-matched). Dotted lines represent the threshold for magnitude of change (X-axis) and significance (Y-axis). (**A**) Volcano plot of differentially expressed genes (DEGs) in unstimulated CD3+IL-1R8+ T-cells in UC samples compared to HCs. (**B**) Volcano plot of DEGs in PMA- and ionomycin-stimulated CD3+IL-1R8+ T-cells in UC samples compared to HCs. Yellow-coloured DEGs are genes related to histone pathways. Blue-coloured DEGs are genes related to mitochondrial energy. Red-coloured DEGs are genes mentioned in the results and discussion. Statistics used are described in Methods under RNAseq analysis.

**Figure 8:**
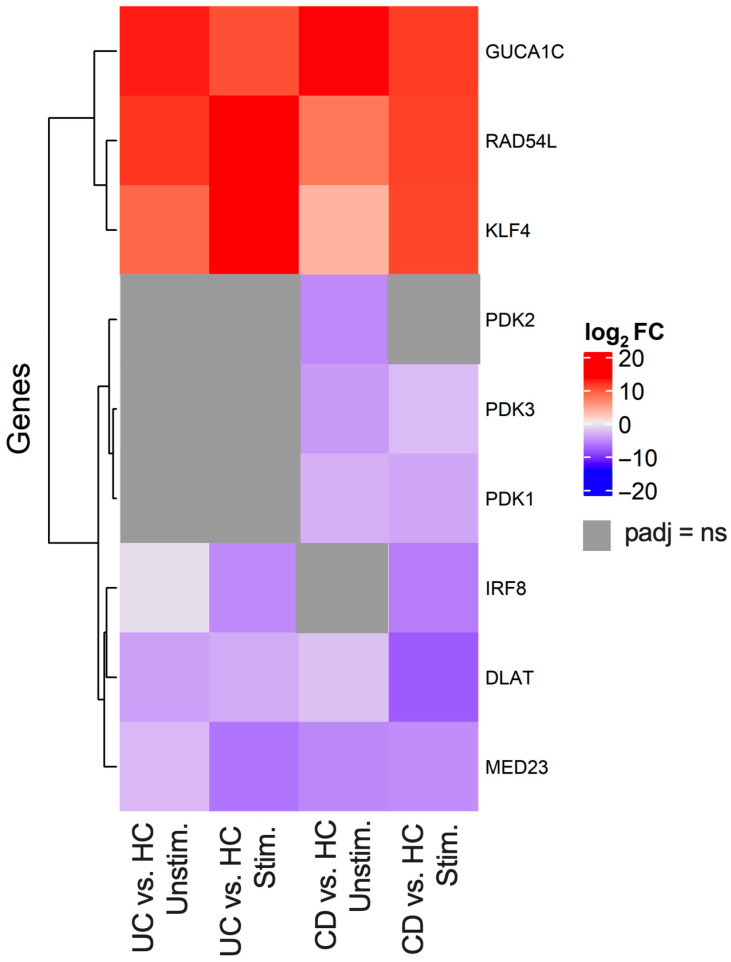
Key differentially expressed genes in CD3+ IL-1R8+ T-cells from IBD PBMCs compared to HCs. Log_2_ fold change (FC) of key differentially expressed genes identified as genes that signify CD3+ IL-1R8+ T-cells from UC and CD patients compared to their expression in CD3+ IL-1R8+ T-cells from HCs. CD3+ IL-1R8+ T-cells were either stimulated with PMA and ionomycin (Stim.) or unstimulated (Unstim).

To further understand the gene expression profiles of T-cells expressing IL-37 receptors in the context of IBD, CD3+ T-cells expressing IL-1R8 were flow cytometry sorted from the PBMCs of IBD patients (CD and UC) and healthy controls, followed by bulk RNA sequencing. Principal component analysis (PCA) demonstrated a clear separation in the IL-1R8+ T-cell RNAseq profiles between HC groups and IBD groups (both UC and CD) as well as a clear separation between stimulated and unstimulated samples, except for one UC donor who was clustered with the HC groups (Supplementary Figure S5). Differentially expressed genes from IL-1R8+ T-cells in IBD signified immune dysregulation indicative of IBD. Differential gene expression analysis between UC and HC from IL-1R8+ CD3+ T-cells revealed the genes most increased in expression in IBD were RAD54L, a gene enriched in Tregs, the histone family genes H2AC16, H2AC13, H2AC25 and H2BC17 as well as KLF4, ZNF608, SORT1, and PFKFB4 (Figure 6, Figure 7 and Figure 8). These genes were most increased in UC and CD patients regardless of stimulation but SORT1, KLF4, PFKFB4 and histone family genes H2BC17 and H2AC16 were increased much more when stimulated compared to unstimulated for CD and UC patients. The main genes most decreased in expression in UC patient stimulated CD3+ IL-1R8+ T-cells were MED23, a gene that mediates autoimmunity, IRF8, a gene that represses IFN signalling, and zinc finger protein genes ZNF7 and ZNF692 (Figure 7 and Figure 8). The same analysis for CD versus HC stimulated samples showed that PAX5 and PXDC1 were key genes with increased expression in CD and not UC (Figure 6).

Genes involved in pathways related to aerobic respiration and respiratory electron transport were significantly differentially expressed in CD patients but not UC patients in both stimulated (*p* = 1.13 × 10^−31^) and unstimulated (*p* = 1.32 × 10^−26^) IL-1R8+ T-cells compared to HCs. More specifically, genes from pathways involved in the regulation of pyruvate metabolism (Stimulated *p* = 1.60 × 10^−5^, Unstimulated *p* = 6.98 × 10^−5^), and regulation of the pyruvate dehydrogenase (PDH) complex (Stimulated *p* = 0.008451624, Unstimulated *p* = 0.0156803) were differentially expressed in CD but not UC patients. Genes signifying mitochondrial dysfunction, such as DLAT and pyruvate dehydrogenase kinase genes PDK1, PDK2 and PDK3 but not PDK4, were genes that were decreased in expression in CD patients (Figure 6, Figure 7 and Figure 8).

Differential gene expression analysis between UC patients and HCs from unstimulated IL-1R8+ CD3+ T-cells revealed the genes most increased in expression in UC were RAD54L, indicating Treg enrichment, and histone family genes H2BC15, H2AC25 and H2AC13. The main genes decreased in expression were FASTK, NELFB, SQLE, LRRC8D, BID, COASY and ZMYM5 (Figure 7 and Figure 8). The same analysis on unstimulated IL-1R8+ T-cells between CD and HC stimulated samples showed PAX5, PXDC1, IRF2BPL, IER5, HES1, ZFP36L1 and TTC7B were key genes increased in expression and HLTF, RINL, ATP2B4, ACAP1, HPS4 and TNFAIP8L1 were key genes decreased in expression (Figure 6). Genes that had increased expression in both CD and UC compared to HCs were GUCA1C from the guanylate cyclase C signalling pathway, histone family gene H3.5, as well as MEIS1, TTC7B and BCL11A.

### 2.4. IL-37 Supplementation May Be Beneficial for Tregs in IBD

Since we identified Tregs as a major source of the anti-inflammatory cytokine IL-37 within PBMC-derived T-cell subsets and with Treg cell therapies or Treg-modulating therapies being explored in IBD, we investigated whether IL-37 supplementation can improve Treg phenotypic stability and Treg suppressive function. To do so, we analysed Treg phenotypes by flow cytometry and performed an in vitro suppression assay.

**Figure 9:**
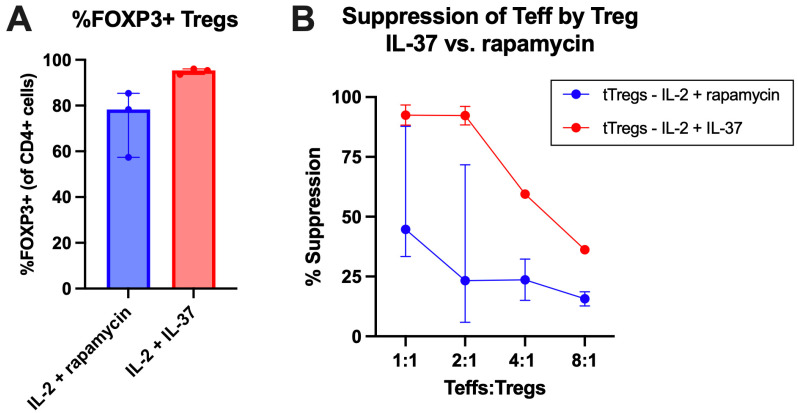
Treatment with IL-37 can maintain Treg phenotype and suppressiveness. (**A**) FOXP3 percentage of the CD4+CD25+ cells of Tregs treated with rapamycin (*n* = 3) and Tregs treated with IL-37 (*n* = 3). (**B**) Suppression of Teffs by Tregs at differing concentrations of Tregs after 5-day suppression assay.

To determine the phenotypic stability of cultured Tregs, we assessed Treg phenotype in the presence of IL-37 or the mammalian target of rapamycin (mTOR) inhibitor rapamycin, which is known to maintain Treg phenotypic stability [46]. The Treg phenotype was measured by expression of FOXP3 on CD4+CD25+ T-cells (Supplementary Figure S6A). We determined that IL-37-treated Tregs were able to maintain FOXP3 expression (mean 95.0% FOXP3+) similar to rapamycin-treated Tregs (mean 73.70% FOXP3+), which is considered the gold-standard [46] (Figure 9A).

To assess the suppression of Teffs by Tregs supplemented with IL-37 or rapamycin, a suppression assay was performed. Teff suppression was assessed according to the dilution of CTV (Figure 9B) to measure suppression Teffs proliferation. IL-37-treated Tregs and Tregs treated with rapamycin both showed a robust suppressor phenotype over Teff proliferation (Figure 9B).

## 3. Discussion

IBD is a heterogeneous disease characterised by chronic inflammation of the GI tract. Tregs play a crucial role in maintaining immune homeostasis in the intestine, and Treg dysfunction has been identified as a key factor contributing to IBD. Despite the array of available treatments, many therapies are ineffective, hence there is an unmet need for novel, safe and effective therapies. Here we identify expression profiles of the anti-inflammatory cytokine IL-37 and its receptors IL-1R5 and IL-1R8 that show dysregulation in IBD. We identify key differentially expressed genes and pathways indicative of IL-1R8+ T-cells in IBD, which signify the immune dysregulation and colonic damage indicative of IBD. We show evidence that IL-37 supplementation can maintain Treg phenotypic stability and suppressive function in vitro. Our data provides previously unknown insight into the expression of IL-37 and its receptors in T-cells in IBD, which could be used as biomarker or therapeutic targets for IBD.

### 3.1. IL-37 Is Differentially Expressed Among PBMC T-Cell Subsets in IBD

We found that IL-37 expression is increased in CD4+ and CD8+ T-cells in CD PBMCs, and of these CD4+ T-cells, we determined increased expression of IL-37 in the circulating Th1 and Th17 cells of CD patients. This is consistent with findings that CD is a predominantly Th1/Th17 cell-driven disease [7,8,9]. The increased IL-37 expression on T-cells in CD patients suggests they are dysfunctional in immunoregulatory mechanisms, as the increased anti-inflammatory IL-37, an attempt to restore immune homeostasis, is insufficient to curtail IBD-related inflammation [47]. Similar observations, where an increase in anti-inflammatory cytokines fails to control inflammation, have been made in other autoimmune conditions, such as systemic lupus erythematosus [48].

Here, we also observed that peripheral blood Th2 cells from IBD patients have increased IL-37 expression compared to HCs. Previous reports suggest Th2 cells upregulate anti-inflammatory cytokines, such as IL-37 and its receptors, in an attempt to regulate and balance the inflammatory response, yet it is often insufficient to curtail IBD-related inflammation [49]. Similar observations have been made for IL-37 in necrotizing enterocolitis, stroke and RA [50] and for other anti-inflammatory cytokines such as IL-38 in SLE [48].

Previous reports indicate that IL-37 is decreased in the serum of IBD patients [42] and this contradictory report may be due to differences in timing and analysis of serum and PBMC sampling. One group found that the percentage of circulating IL-37+ monocytes, natural killer, and B cells increased in IBD patients compared with HCs [51]. We report here also an increase in IL-37 in T-cells in IBD patients, which implicates IL-37+ T-cells in the dysregulated phenotype seen in IBD.

When keeping disease state steady and comparing the expression of IL-37 and its receptors from each cell type, the expression of IL-37 was significantly higher in Treg cells compared to Th1 cells, supporting previous findings that Tregs express IL-37 [35,36].

### 3.2. The IL-37 Receptor, IL-1R5 and IL-1R8, Are Differentially Expressed in PBMC-Derived T-Cells in IBD

We analysed T-cells double positive for IL-1R5 and IL-1R8, as it has been shown that IL-37 is required for the assembly of a tripartite complex, comprising IL-37, IL-1R5 and IL-1R8, for its anti-inflammatory function [34]. Similarly to IL-37 expression, we found that co-expression of IL-1R5 and IL-1R8 was increased in the Th1 and Th17 cells of CD patients, further solidifying the conclusion that these cells can bind to extracellular IL-37. The increase in the abundance of IL-1R5 and IL-1R8 in Treg cells from CD patients points to an anti-inflammatory regulatory mechanism to counteract the heightened pro-inflammatory responses seen in CD that is aimed to restore homeostasis. UC has been suggested to be a Th2-driven disease [10,52], however, we found no increase in the expression of IL-37 receptors in Th2 cells in UC compared to HCs. This finding may indicate that these cells are less responsive to IL-37 and may therefore be dysfunctional in dampening uncontrolled inflammation.

Additionally, we observed a decrease in the abundance of IL-1R5 and IL-1R8 receptor expression in cells stimulated with PMA and ionomycin, which may be the result of IL-1R8 internalisation in activated T-cells [53]. Furthermore, it has been reported that glycogen synthase kinase-3β (GSK3β) can phosphorylate IL-1R8 in lung epithelial cells [54], leading to IL-1R8 internalisation. It is also known that PMA, which we used for T-cell stimulation, can increase the function of GSK3β, resulting in increased internalisation, which was shown in mesenchymal stem cells [55].

Our flow cytometry results here reflect the previous findings determined by immunoblotting, indicating that human Treg cells express IL-37 [35]. Our results indicate that despite the increase in co-expression of IL-1R5 and IL-1R8 in CD, there is no difference in Treg IL-37 abundance between HC and IBD patients. As IBD patients are experiencing inflammation, it would be expected that IL-1R5 and IL-1R8 would be upregulated to counterbalance the inflammation. In CD, the lack of IL-37 receptor upregulation might be a contributing factor to Treg dysfunction, since Treg-derived IL-37 may not be able to adequately signal via its receptors on Tregs, contributing to Treg dysfunction seen in CD.

Previous reports have shown that IL-1R8 is not expressed in Th1 cells using murine models and immunoblots [49,56]. However, our flow cytometry results from human samples show human Th1 cells do express IL-1R8, which could be due to a difference between species.

### 3.3. LPMC-Derived IL-37 Is Elevated in IBD CD8+ T-Cells, While Its Receptor Is Decreased in IBD CD4+ T-Cells

Here we show for the first time that LPMCs from IBD patients express IL-37, IL-1R5 and IL-1R8. It was previously known that IL-37 is upregulated in the colon of IBD patients [42]. We show here that IL-37 expression was increased in CD8+ T-cells in both CD and UC patients compared to HCs, highlighting an attempt to restore immune homeostasis. However, we found that IL-1R5 and IL-1R8 co-expression was decreased in LPMC CD4+ T-cells in CD patients. Despite the increase in IL-37 expression on LPMC T-cells, in CD, a lack of receptor expression may inhibit the anti-inflammatory function of IL-37, facilitating uncontrolled inflammation. This could also be due to internalization and subsequent phosphorylation of the IL-1R5 and IL-1R8 receptors upon engagement with IL-37 [57]. It is known that when IL-1R5 and IL-1R8 receptors bind soluble IL-37, the receptors are internalized and degraded via the ubiquitin protease system [54,58,59,60]. This forms a key negative regulator of inflammation. Therefore, in active IBD patients, at the site of disease where the LPMCs are present, the downregulation of surface expression of the IL-1R5 and IL-1R8 receptors may be due to engagement with soluble IL-37 and therefore signify an attempt by the CD4+ T-cells in the gut to reduce inflammation at the site of colonic disease, which is not seen in peripheral blood.

### 3.4. IL-1R8+ T-Cell Gene Signature Reveals Hallmarks of IBD

Expression profile analysis of differentially expressed genes from IL-1R8+ T-cells in UC, CD and HCs revealed genes and pathways already implicated in IBD and novel associations with IBD. PCA analysis demonstrated the differential expression of IL-1R8+ T-cells between HC and IBD patients. One of the three UC donors clustered with HC samples, which could be due to individual patient characteristics such as disease severity or active vs. remission state. It is therefore prudent to interpret the UC data with this in mind.

RAD54L is highly differentially upregulated in CD and UC IL-1R8+ T-cells compared to HCs, irrespective of being stimulated or unstimulated. Among immune cell subsets, RAD54L expression is enhanced in Tregs [61], which supports our finding that Tregs were the predominant source of IL-37 receptor expression among T-cells in IBD. RAD54L is involved in DNA damage and DNA repair. In pro-inflammatory conditions, there is an increase in DNA damage requiring repair and therefore the upregulation of RAD54L in UC and CD may be a repair response to damage the Treg cell has encountered from a more pro-inflammatory environment compared to that of HC. Interestingly, RAD54L and IL-37 have been identified before as biomarkers in distinguishing different forms of contact dermatitis [62]. This highlights that upregulation of RAD54L could be predictive of certain inflammatory disease states, including in IBD and regardless of stimulation state.

KLF4 is differentially upregulated in CD and UC IL-1R8+ T-cells compared to HCs when stimulated. In HCs, KLF4 is not typically expressed on T-cells [61]. However, in chronic inflammatory conditions, KLF4 is known to be associated with exhausted CD8+ T-cells [63]. In the context of IBD, IL-37 has been shown to promote colitis-associated colon cancer through IL-1R8-dependent CD8+ T-cell dysfunction [37]. Flow cytometry data showed the percentages of stimulated IL-1R8+ CD8+ T-cells between IBD and HCs remained relatively constant. This highlights that it is not a difference in total RNA from increased CD8+ T-cell numbers causing the increased expression, but rather an increase in KLF4 expression in IBD IL-1R8+ T-cells, likely from exhausted CD8+ T-cells. Thus, KLF4 may indicate CD8+ T-cell dysfunction in IBD

H2AC16, synonymous with H2AC11, H2AC13, H2AC16 and H2AC17, encodes histone H2A type 1 and is significantly upregulated in IL-1R8+ T-cells in both UC and CD compared to HCs, regardless of stimulation state. Upregulation of this gene was previously found in the inflamed colonic mucosa of patients with CD compared to HCs [64]. The upregulation of H2AC16 was also found to be positively correlated with inflammatory characteristics indicative of CD, such as C reactive protein (CRP), Crohn’s disease activity index (CDAI), simple endoscopic score-CD (SES-CD) and global histology activity score (GHAS). Given that H2AC16 is associated with inflammation in CD and found in the colonic mucosa, our findings show that IL-1R8+ T-cells are a source of H2AC16 and could be indicative of IBD.

Other histone proteins (H2AC25 and H3.5) were found to be upregulated in CD in both stimulated and unstimulated conditions. Dysregulation in epigenetic modifications around the way the nucleosome packages chromatin affects transcription regulation, and the differential expression of chromatin-related genes has been identified in IBD [65]. This can explain the many upregulated histone H2A, H2B and H3 genes we observed as upregulated in IBD IL-1R8+ T-cells compared to HCs, which points to a transcription signature enriched in H2A, H2B and H3 genes as being indicative of IBD. Further research is needed to uncover the mechanistic role of histone modification in IBD.

The most downregulated gene in stimulated IL-1R8+ T-cells in UC compared to HCs is MED23. This gene contributes to controlling T-cell activation and preventing autoimmunity [66]. Specifically, MED23 negatively regulates T-cell activation, and MED23 deficient mice showed hyperactive T-cells and autoimmune syndrome. Therefore, its downregulation in UC could be a hallmark of inflammatory disease in IL-1R8+ T-cells in UC.

A key downregulated gene in stimulated UC IL-1R8+ T-cells compared to HC is IRF8, which encodes interferon (IFN) regulatory factor 8, a transcription factor that represses IFN-stimulated response elements [67]. Since IL-1R8+ T-cells are stimulated and UC samples downregulate it, this suggests there is less ability to repress IFN family gene expression in UC. This highlights the dysregulation of IL-1R8+ T-cells in UC, as in the face of proinflammatory stimulus they are unable to regulate IFN gene expression compared to HCs, which likely leads to heightened inflammation contributing to UC.

Genes involved in aerobic respiration and respiratory electron transport and specifically in the regulation of pyruvate metabolism and pyruvate dehydrogenase (PDH) complex were differentially expressed in CD IL-1R8+ T-cells compared to HC. The regulation of T-cell metabolic machinery is crucial for T-cell immune function and T-cells specifically use glycolysis when activated so that they can adequately respond to the extra metabolic demands of the activated state [68]. Our data show genes from these pathways are differentially expressed in CD but not UC IL-1R8+ T-cells, which could be a cause of or response to the hyper-inflammatory immune state seen in T-cells from CD patients. Indeed, another study has shown that modulating CD4+ T-cell PIK4, a gene involved in the PDH complex, in a DSS-induced mouse model could mitigate colitis, suggesting such genes could be a target for CD [69]. Interestingly, PIK4 was not differentially expressed in CD IL-1R8+ T-cells in our dataset. However, other pyruvate dehydrogenase kinase genes (PIK1, PIK2 and PIK3) were all downregulated in CD IL-1R8+ T-cells compared to HCs.

Of note, DLAT was the most downregulated gene in stimulated IL-1R8+ T-cells in CD. It is involved in the regulation of pyruvate metabolism and the PDH complex. It is also involved in cuproptosis, which is a form of cell death linked to mitochondria respiration that has been implicated in IBD [70,71].

The most upregulated gene in unstimulated IL-1R8+ T-cells in CD and UC is GUCA1C, which encodes guanylate cyclase activator 1C. The guanylate cyclase-C signalling pathway is known to be dysregulated in IBD and associated with abdominal pain, constipation, diarrhea, dysfunctional epithelial barrier function, polyps and colorectal cancer [72,73]. Other genes involved in this process have been implicated in IBD, namely GUCY2C and GUCA2B. Our novel observation that GUCA1C is most upregulated in IL-1R8+ T-cells from IBD patients further highlights the role of the guanylate cyclase-C signalling pathway in the pathogenesis of IBD.

We analysed total IL-1R8+ CD3+ T-cells for their RNA transcriptomic signature and therefore are unable to resolve the contribution of the various T-cell subsets in this study. For each gene, it is possible that a particular IL-1R8+ T-cell subset is predominantly contributing to the differential gene expression between healthy and IBD patients. More detailed studies utilizing single-cell RNA sequencing focusing on the expression of the genes we have identified here within T-cell subsets are needed to fully understand which IL-1R8+ T-cells are implicated in IBD.

Given that our results are only transcriptomic, further research is needed to validate each gene for its protein expression as well as assessing whether the mitochondrial and metabolic genes identified here affect IL-1R8+ T-cell function.

The differential expression of the genes uncovered here provides a snapshot of the gene expression patterns in IL-1R8+ T-cells in IBD patients. Given that only three individuals were analysed per group, further research is needed to fully capture the heterogeneity of IBD patients and mechanistic studies will be necessary to elucidate how the genes identified here are associated with disease severity and patient outcomes.

### 3.5. IL-37 Supplementation May Be Beneficial for Tregs in IBD

The cause of Treg dysfunction in IBD is likely to be multifactorial including anergy, plasticity, exhaustion and the diminished ability to traffic to the inflamed gut [17,23]. Tregs may also undergo apoptosis more frequently in the LP of IBD patients [22]. Treg cell therapy has also been explored to treat IBD in clinical trials aimed at overcoming the Treg dysfunction seen in IBD [74].

Here, we supplemented cultures with IL-37, as described in [75]. We determined that when culturing Tregs with IL-37, FOXP3 expression and Teff suppression were maintained, and these effects were at least as pronounced as when the gold-standard in Treg stabilisation assays, rapamycin, was used. It has been shown previously that IL-37b-transfected cells reduced the expression of mTOR, showing that IL-37 can function as an mTOR inhibitor, similar to rapamycin, thus stabilising the Treg phenotype [31]. In vitro supplementation with rapamycin has been shown to enhance the suppressive capacity of cultured Tregs [76], while mouse models have shown effectiveness in IBD [77]. Here, we present evidence that IL-37 can maintain Treg phenotype and function, similar to an already accepted Treg-stabilising in vitro culture reagent, rapamycin. Given the small sample size in the suppression assay, further research with a greater sample size is needed to assess the ability of IL-37 to enhance Treg phenotype and function compared to rapamycin.

There has been increasing interest in Treg therapy for the treatment of autoimmune diseases, including IBD [74,78]. The efficacy of Treg therapy may be enhanced by the addition of IL-37 as a combination therapy or IL-37 alone may be used to promote Treg function.

### 3.6. The Context-Specific Effects of IL-37 in IBD

The use of IL-37 as a biologic treatment for IBD requires careful consideration of its context-specific effects. Particularly, understanding the balance between the anti-inflammatory effect of IL-37 and its potential to promote cancers like colorectal cancer is an important consideration, as anti-inflammatory molecules can also reduce immune response against cancer [37]. Also, it is important to consider the microbiome when developing IL-37 as a biologic treatment for IBD, as mouse models have shown that the anti-inflammatory effect of IL-37 is dependent on a healthy microbiome [41].

## 4. Methods

### 4.1. Informed Consent Statement

All procedures were performed in accordance with the Declaration of Helsinki. All study participants provided written informed consent. Ethics approval was obtained from the Monash Health Human Research Ethics Committee and approved on 5 April 2016 (reference 16095A).

### 4.2. Patient Sample Collection

Peripheral blood and colonic biopsy samples were collected from non-IBD controls and IBD patients (CD and UC) attending the Department of Gastroenterology at Monash Medical Centre, Monash Health, Clayton, Australia. All IBD patients had a previous diagnosis prior to collection. Non-IBD controls (healthy control (HC)) were only included after confirmation that they had no autoimmune disease, no current active infection at the time of sample collection and were not on immunomodulating or immunosuppressive treatment.

### 4.3. Peripheral Blood Mononuclear Cell Isolation

Peripheral whole blood was diluted 1:2 in phosphate-buffered saline (PBS, Gibco, Waltham, MA, USA) and PBMCs were isolated by density-gradient centrifugation over Histopaque-1077 (Sigma-Aldrich, St. Louis, MO, USA) in SepMate PBMC isolation tubes (Stemcell, Vancouver, BC, Canada). After isolation, PBMCs were resuspended in RPMI 1640 (Gibco, Waltham, MA, USA) supplemented with 1% human serum from male, AB, clotted whole blood (Sigma-Aldrich, St. Louis, MO, USA) and 1:500 MycoZap Plus-PR (Lonza, Basel, Switzerland) and seeded at a density of 1 × 10^6^ cells/mL for flow cytometry for immunophenotyping experiments and incubated at 37 °C, 5% CO_2_ for 1 h.

### 4.4. Lamina Propria Mononuclear Cell Isolation

Colonic biopsies were collected in Hank’s Balanced Salt Solution (HBSS, Sigma-Aldrich, St. Louis, MO, USA) and were washed in HBSS containing 1 mM EDTA with agitation for 10 min at 37 °C. Samples were then digested with 1 mg/mL collagenase Ia (Sigma-Aldrich, St. Louis, MO, USA) and 10 IU/mL DNase I (Roche, Basel, Switzerland) with agitation for 30 min at 37 °C. Following digestion, cells were passed through a 100 µm cell strainer. Lamina propria mononuclear cells (LPMCs) were then seeded into 2 wells in a 48-well flat-bottom plate and incubated at 37 °C, 5% CO_2_ for 1 h.

### 4.5. Stimulation of PBMCs and LPMCs

Following 1 h incubation at 37 °C and 5% CO_2_, PBMCs and LPMCs were either left unstimulated or stimulated with 50 ng/mL phorbol 12-myristate 13-acetate (PMA), 750 ng/mL ionomycin and 10 µg/mL brefeldin-A (all Sigma-Aldrich, St. Louis, MO, USA) for 4 h.

### 4.6. Flow Cytometry for Immunophenotyping

After 4 h stimulation, unstimulated and stimulated samples were washed in PBS and divided across the 4 flow cytometry panels to assess Th1, Th2, Th17 and Treg cells. The antibodies used for flow cytometry are described in Table 2.

All staining steps were performed at room temperature in the dark. Live/dead staining was performed for 20 min. Cells were then re-suspended in PBS and centrifuged at 300× *g* for 5 min followed by cell surface antibody staining for 25 min in the surface antibody cocktail from Table 1 in PBS supplemented with 2% foetal calf serum (FCS) and 1 mM EDTA (FCS/PBS). All samples were then re-suspended in FCS/PBS and centrifuged at 300× *g* for 5 min followed by fixation and permeabilization using a Foxp3/Transcription Factor Staining Buffer Set (eBioscience, Waltham, MA, USA) according to the manufacturer’s instructions. Cells were then stained with intracellular antibody panels 1–4 (Table 1) for 30 min in permeabilization buffer (eBioscience, Waltham, MA, USA). After intracellular staining, cells were centrifuged at 300× *g* for 5 min followed by resuspension in PBS. Experiments were acquired using the BD Fortessa X-20 running FACSDiva Version 9.7 software (Franklin Lakes, NJ, USA) and Cytek Aurora 5 L running SpectroFlo 3.3.0 software (Fremont, CA, USA). Flow cytometry data were analysed with FlowJo v.10.10.0 (Ashland, OR, USA) and GraphPad Prism v.10.4.1 (San Diego, CA, USA).

### 4.7. Sample Preparation for RNAseq

Isolated PBMCs from CD (*n* = 3), UC (*n* = 3) and age- and sex-matched HC (*n* = 6) were rested for 1 h prior to treatment in RPMI 1640 (Gibco) supplemented with 1% human serum from male, AB, clotted whole blood (Sigma-Aldrich) and 1:500 MycoZap Plus-PR (Lonza) (unstimulated) or 16 h stimulation with 2 ng/mL PMA, 250 ng/mL ionomycin and 2 µg/mL brefeldin-A (all Sigma-Aldrich). Unstimulated and stimulated PBMCs were then washed prior to cell surface staining for 25 min at room temperature with CD3 APC/Cyanine7 (clone UCHT1, Tonbo, San Diego, CA, USA) and IL-1R8 Qdot 655 (clone A-4, Santa Cruz, Dallas, TX, USA). Dead cells were excluded using 0.1 µg/mL DAPI (Sigma-Aldrich, St. Louis, MO, USA). Fluorescence-activated cell sorting (FACS) was performed using BD FACS Aria Fusion to sort CD3+IL-1R8+ cells into RNALater (Sigma-Aldrich, St. Louis, MO, USA).

RNA was isolated using an ISOLATE II RNA Mini Kit (Bioline, Cincinnati, OH, USA) according to the manufacturer’s instructions. Quantification and assessment of RNA integrity were determined with an Agilent Bioanalyser using an RNA 6000 Pico kit (Santa Clara, CA, USA) and samples had a mean RNA integrity score of 7.05. RNA-Seq was performed on a NextSeq2000 using a P3 50-cycle kit (Illumina, San Diego, CA, USA).

### 4.8. RNAseq Analysis

Single-end reads (FASTQ) for each sample were imported to the Galaxy Australia [79] web platform and were assessed using FastQC [80] (version 0.12.1) and MultiQC [81] (version 1.11). Reads were trimmed of their 3′ adaptors (poly-A (10)) (Cutadapt) [82] and trimmed reads were aligned to the reference genome hg38 (human) with HISAT2 [83]. Counts were generated using the featureCounts tool [84] on the aligned reads and single-count files were joined to generate a counts matrix. Downstream visualisation and statistical analysis were conducted using R [85] (4.4.2, Vienna, Austria) and RStudio (version 2024.09.1+394, Boston, MA, USA). Genes were retained in a counts matrix if there were at least 3 samples with >10 counts. Gene normalization and differential gene expression between groups were performed using DESeq2 [86] with the apeglm (v3.2) 48 model for shrinking log2 fold changes and the default Wald statistical test. Genes were considered significantly changed if adjusted *p* values were <0.05 and had a log_2_ fold change of ≥|0.58|. Volcano plots were visualised using EnhancedVolcano [87]. For principal component analysis (PCA), raw count data were variance stabilised using variance stabilising transformation (VST) implemented in DESeq2 and the transformed data visualised.

### 4.9. In Vitro Treg Cell Culture

PBMCs were isolated from healthy control buffy coats (Life Blood) approved by the Monash University Human Research Ethics Committee, number 18277. To isolate PBMCs, buffy coats underwent density gradient separation using Lymhoprep (Stemcell) in SepMate tubes (Stemcell) following the manufacturer’s instructions (Stemcell, Vancouver, BC, Canada). CD4+CD127lowCD25+ Tregs and CD4+CD25– T effector cells (Teffs) were isolated from PBMCs using the EasySep Human CD4+CD127lowCD25+ Regulatory T-cell Isolation Kit (Stemcell Vancouver, BC, Canada). Tregs were reconstituted in X-VIVO 15 media (Lonza, Basel, Switzerland) supplemented with 10% human serum (Sigma-Aldrich) and seeded at a density of 0.5 × 10^6^ cells/mL in a 96-well flat-bottom plate. Human T-Activator CD3/CD28 Dynabeads (Gibco, Waltham, MA, USA) were added at a 1:1 ratio to the number of cells plated and incubated at 37 °C and 5% CO_2_. Cells were supplemented with 1000 IU/mL IL-2 (Stemcell, Vancouver, BC, Canada) every 48 h until the end of culture at day 11.

CD4+CD25–T effector cells (Teffs) isolated with an EasySep Human CD4+CD127lowCD25+ Regulatory T-cell Isolation Kit (Stemcell) on the same day of Treg isolation were cryopreserved in FCS containing 10% DMSO until required for suppression assay set-up.

### 4.10. Suppression Assay Co-Culture

Teffs isolated from the EasySep Human CD4+CD127lowCD25+ Regulatory T-cell Isolation Kit (Stemcell) as mentioned above were thawed and rested overnight prior to suppression assay set-up in X-VIVO 15 media (Lonza). Teffs were prepared for the suppression assay by labelling with proliferation dye CellTrace Violet (CTV, Invitrogen, Waltham, MA, USA) for 3 min at room temperature in the dark. The reaction was quenched with 2% FCS and the cells were washed in X-VIVO 15 media twice. Tregs used in the suppression assay were obtained after an in vitro expansion step outlined in the ‘in vitro Treg cell culture’ section above.

Suppression assay co-culture set-up with Teffs and Tregs, and calculation of percentage suppression was performed, as previously described [23]. Briefly, CTV-labelled Teffs were cultured with serial dilutions of in vitro-expanded Tregs in the presence of 1000 IU/mL IL-2 (Stemcell) and either IL-37 protein or rapamycin (Merck, St. Louis, MO, USA). 1000 IU/mL IL-2 (Stemcell) was added to all wells on the day of co-culture setup and then re-supplemented every 2 days of culture. Recombinant human IL-3746–218 Y85A (IL-37) was produced, as described previously [75]. 100 pg/mL IL-37 was added to the IL-37 treated group on the day of co-culture setup. The rapamycin-treated group received 100 nM rapamycin (Merck) on the day of co-culture setup. Suppression assay was assessed by flow cytometry Panel 4 (Table 1) after 5 days of incubation at 37 °C, 5% CO_2_.

### 4.11. Statistical Analysis

Statistical analysis was performed using GraphPad Prism 9 (San Diego, CA, USA). Normality was determined using the Shapiro–Wilk test. All data was non-normally distributed. For isolated PBMCs and LPMCs, Mann–Whitney U tests were performed to determine significance for each data group. Outliers were removed using the ROUT outliers test. A *p* value < 0.05 was considered statistically significant.

## 5. Conclusions and Future Directions

We have explored the expression of IL-37 and its receptors IL-1R5 and IL-1R8 on T-cells and uncovered differences in CD and UC patient T-cells compared to healthy controls that may underpin immune dysregulation seen in IBD. We have further explored the immune dysregulation in IL-1R8+ T-cells, identified differentially expressed genes and identified pathways, such as histone and mitochondrial pathways, that can explain how IBD patients’ T-cells are recalcitrant in maintaining gut immune homeostasis. Finally, we have explored the use of recombinant IL-37 supplementation and its effects on T-cells and Tregs, showing improvement in Treg function with IL-37 supplementation in vitro. These findings together uncover the different biology of IL-37 and its receptors on T-cells in IBD, which can be utilised for targeted immune therapies to restore the T-cell imbalance driving IBD pathology.

Although differences were observed between IBD patients and healthy controls, the small sample size is a limitation of the study, as it may not adequately capture the heterogeneity seen in IBD patients. Future studies with greater sample sizes can build on our findings to fully capture the phenotypes of T-cells expressing IL-37 and its receptors in IBD.

The next steps in this research will involve validating the key genes identified in transcriptomic profiling for their protein expression and functional effects on IL-1R8+ T-cells, as well as identifying the T-cell subsets responsible for differential gene expression.

## Figures and Tables

**Table 1:** Demographics of IBD patients and healthy controls (HC) included in the study.

Characteristic	Value
**HC**	18
Age (years), median (range)	50 (23–75)
Female sex, n (%)	11 (61%)
Biopsies obtained, n (%)	10 (55%)
Blood obtained, n (%)	18 (100%)
**CD Patients**	15
Age (years), median (range)	27 (19–62)
Female sex, n (%)	6 (40%)
Medical therapy, n (%)	
Biologics:	
Infliximab	4 (26%)
Adalimumab	4 (26%)
Ustekinumab	3 (20%)
5-aminosalicylic acid Corticosteroids	2 (13%) 1 (6.7%)
Other immunomodulators	3 (20%)
No therapy	2 (13%)
Biopsies obtained, n (%)	10 (67%)
Evidence of mucosal inflammation, n (%) Blood obtained, n (%)	3 (30%) 15 (100%)
**UC Patients**	14
Age (years), median (range)	38 (21–58)
Female sex, n (%)	7 (50%)
Medical therapy, n (%)	
Biologics:	
Infliximab	3 (21%)
Vedolizumab	2 (14%)
5-aminosalicylic acid	9 (64%)
Other immunomodulators Unreported	5 (35%) 1 (7.1%)
Biopsies obtained, n (%)	10 (71%)
Evidence of mucosal inflammation, n (%) Blood obtained, n (%)	5 (50%) 14 (100%)

Abbreviations: HC—healthy control; CD—Crohn’s disease; UC—ulcerative colitis.

**Table 2:** Antibodies used in flow cytometry.

Specificity	Clone	Fluorochrome	Source	Catalogue Number
**All panels**				
Live/Dead **Surface Antibodies:**	N/A	Fixable Viability Stain 780	BD	565388
CD4	SK3 (Leu3a)	BUV395	BD	563550
CD8	SK1	Alexa Fluor 700	BioLegend	344724
IL-1R5 (IL-18Rα)	70625	Alexa Fluor 488	R&D	FAB840G
IL-1R8 (SIGIRR)	A-4	Qdot 655 with SiteClick Antibody Labelling Kit #S10453 (Invitrogen)	Santa Cruz	sc-271864
IL-37	37D12	PE	eBioscience	12-7379-42
**Panel 1 (Th1): Intracellular**				
T-bet	eBio4B10 (4B10)	ef660	eBioscience	50-5825-82
**Panel 2 (Th2): Intracellular**				
GATA3	TWAJ	ef660	eBioscience	50-9966-42
**Panel 3 (Th17): Intracellular**				
RORγt	AFKJS-9	APC	eBioscience	17-6988-82
**Panel 4 (Tregs): Intracellular**				
FOXP3	PCH101	APC	eBioscience	17-4776-42

Abbreviations: APC—Allophycocyanin; BUV—Brilliant Ultra Violet; PE—Phycoerythrin; Qdot—Quantum dot.

## Data Availability

The data underlying this article will be shared on reasonable request to the corresponding author.

## Supplementary Materials

The following supporting information can be downloaded at: https://www.mdpi.com/article/10.3390/ijms27031540/s1.

## References

[1] Ng S.C., Shi H.Y., Hamidi N., Underwood F.E., Tang W., Benchimol E.I., Panaccione R., Ghosh S., Wu J.C.Y., Chan F.K.L. (2017). Worldwide incidence and prevalence of inflammatory bowel disease in the 21st century: A systematic review of population-based studies. Lancet.

[2] Vieujean S., Jairath V., Peyrin-Biroulet L., Dubinsky M., Iacucci M., Magro F., Danese S. (2025). Understanding the therapeutic toolkit for inflammatory bowel disease. Nat. Rev. Gastroenterol. Hepatol..

[3] Chang J.T. (2020). Pathophysiology of Inflammatory Bowel Diseases. N. Engl. J. Med..

[4] Schultsz C., Van Den Berg F.M., Ten Kate F.W., Tytgat G.N., Dankert J. (1999). The intestinal mucus layer from patients with inflammatory bowel disease harbors high numbers of bacteria compared with controls. Gastroenterology.

[5] Prorok-Hamon M., Friswell M.K., Alswied A., Roberts C.L., Song F., Flanagan P.K., Knight P., Codling C., Marchesi J.R., Winstanley C. (2014). Colonic mucosa-associated diffusely adherent afaC+ Escherichia coli expressing lpfA and pks are increased in inflammatory bowel disease and colon cancer. Gut.

[6] Takaishi H., Matsuki T., Nakazawa A., Takada T., Kado S., Asahara T., Kamada N., Sakuraba A., Yajima T., Higuchi H. (2008). Imbalance in intestinal microflora constitution could be involved in the pathogenesis of inflammatory bowel disease. Int. J. Med. Microbiol..

[7] Parronchi P., Romagnani P., Annunziato F., Sampognaro S., Becchio A., Giannarini L., Maggi E., Pupilli C., Tonelli F., Romagnani S. (1997). Type 1 T-helper cell predominance and interleukin-12 expression in the gut of patients with Crohn’s disease. Am. J. Pathol..

[8] Niessner M., Volk B.A. (1995). Altered Th1/Th2 cytokine profiles in the intestinal mucosa of patients with inflammatory bowel disease as assessed by quantitative reversed transcribed polymerase chain reaction (RT-PCR). Clin. Exp. Immunol..

[9] Schmidt C., Giese T., Ludwig B., Mueller-Molaian I., Marth T., Zeuzem S., Meuer S.C., Stallmach A. (2005). Expression of interleukin-12-related cytokine transcripts in inflammatory bowel disease: Elevated interleukin-23p19 and interleukin-27p28 in Crohn’s disease but not in ulcerative colitis. Inflamm. Bowel Dis..

[10] Fuss I.J., Heller F., Boirivant M., Leon F., Yoshida M., Fichtner-Feigl S., Yang Z., Exley M., Kitani A., Blumberg R.S. (2004). Nonclassical CD1d-restricted NK T cells that produce IL-13 characterize an atypical Th2 response in ulcerative colitis. J. Clin. Invest..

[11] Wang Y., Liu X.P., Zhao Z.B., Chen J.H., Yu C.G. (2011). Expression of CD4+ forkhead box P3 (FOXP3)+ regulatory T cells in inflammatory bowel disease. J. Dig. Dis..

[12] Maul J., Loddenkemper C., Mundt P., Berg E., Giese T., Stallmach A., Zeitz M., Duchmann R. (2005). Peripheral and intestinal regulatory CD4+ CD25(high) T cells in inflammatory bowel disease. Gastroenterology.

[13] Hori S., Nomura T., Sakaguchi S. (2003). Control of regulatory T cell development by the transcription factor Foxp3. Science.

[14] Fontenot J.D., Gavin M.A., Rudensky A.Y. (2003). Foxp3 programs the development and function of CD4(+)CD25(+) regulatory T cells. Nat. Immunol..

[15] Vignali D.A., Collison L.W., Workman C.J. (2008). How regulatory T cells work. Nat. Rev. Immunol..

[16] Hadis U., Wahl B., Schulz O., Hardtke-Wolenski M., Schippers A., Wagner N., Muller W., Sparwasser T., Forster R., Pabst O. (2011). Intestinal tolerance requires gut homing and expansion of FoxP3+ regulatory T cells in the lamina propria. Immunity.

[17] Hovhannisyan Z., Treatman J., Littman D.R., Mayer L. (2011). Characterization of interleukin-17-producing regulatory T cells in inflamed intestinal mucosa from patients with inflammatory bowel diseases. Gastroenterology.

[18] Takahashi M., Nakamura K., Honda K., Kitamura Y., Mizutani T., Araki Y., Kabemura T., Chijiiwa Y., Harada N., Nawata H. (2006). An inverse correlation of human peripheral blood regulatory T cell frequency with the disease activity of ulcerative colitis. Dig. Dis. Sci..

[19] Eastaff-Leung N., Mabarrack N., Barbour A., Cummins A., Barry S. (2010). Foxp3+ regulatory T cells, Th17 effector cells, and cytokine environment in inflammatory bowel disease. J. Clin. Immunol..

[20] Li J., Ueno A., Iacucci M., Fort Gasia M., Jijon H.B., Panaccione R., Kaplan G.G., Beck P.L., Luider J., Barkema H.W. (2017). Crossover Subsets of CD4(+) T Lymphocytes in the Intestinal Lamina Propria of Patients with Crohn’s Disease and Ulcerative Colitis. Dig. Dis. Sci..

[21] Lord J.D., Shows D.M., Chen J., Thirlby R.C. (2015). Human Blood and Mucosal Regulatory T Cells Express Activation Markers and Inhibitory Receptors in Inflammatory Bowel Disease. PLoS ONE.

[22] Veltkamp C., Anstaett M., Wahl K., Moller S., Gangl S., Bachmann O., Hardtke-Wolenski M., Langer F., Stremmel W., Manns M.P. (2011). Apoptosis of regulatory T lymphocytes is increased in chronic inflammatory bowel disease and reversed by anti-TNFalpha treatment. Gut.

[23] Goldberg R., Scotta C., Cooper D., Nissim-Eliraz E., Nir E., Tasker S., Irving P.M., Sanderson J., Lavender P., Ibrahim F. (2019). Correction of Defective T-Regulatory Cells From Patients With Crohn’s Disease by Ex Vivo Ligation of Retinoic Acid Receptor-alpha. Gastroenterology.

[24] Fantini M.C., Rizzo A., Fina D., Caruso R., Sarra M., Stolfi C., Becker C., Macdonald T.T., Pallone F., Neurath M.F. (2009). Smad7 controls resistance of colitogenic T cells to regulatory T cell-mediated suppression. Gastroenterology.

[25] Boraschi D., Lucchesi D., Hainzl S., Leitner M., Maier E., Mangelberger D., Oostingh G.J., Pfaller T., Pixner C., Posselt G. (2011). IL-37: A new anti-inflammatory cytokine of the IL-1 family. Eur. Cytokine Netw..

[26] Luo Y., Cai X., Liu S., Wang S., Nold-Petry C.A., Nold M.F., Bufler P., Norris D., Dinarello C.A., Fujita M. (2014). Suppression of antigen-specific adaptive immunity by IL-37 via induction of tolerogenic dendritic cells. Proc. Natl. Acad. Sci. USA.

[27] Allaire J.M., Poon A., Crowley S.M., Han X., Sharafian Z., Moore N., Stahl M., Bressler B., Lavoie P.M., Jacobson K. (2021). Interleukin-37 regulates innate immune signaling in human and mouse colonic organoids. Sci. Rep..

[28] Teufel L.U., Matzaraki V., Folkman L., Ter Horst R., Moorlag S., Mulders-Manders C.M., Netea M.G., Krausgruber T., Joosten L.A.B., Arts R.J.W. (2024). Insights into the multifaceted role of interleukin-37 on human immune cell regulation. Clin. Immunol..

[29] Sullivan G.P., Davidovich P., Munoz-Wolf N., Ward R.W., Hernandez Santana Y.E., Clancy D.M., Gorman A., Najda Z., Turk B., Walsh P.T. (2022). Myeloid cell-derived proteases produce a proinflammatory form of IL-37 that signals via IL-36 receptor engagement. Sci. Immunol..

[30] Sharma S., Kulk N., Nold M.F., Graf R., Kim S.H., Reinhardt D., Dinarello C.A., Bufler P. (2008). The IL-1 family member 7b translocates to the nucleus and down-regulates proinflammatory cytokines. J. Immunol..

[31] Nold M.F., Nold-Petry C.A., Zepp J.A., Palmer B.E., Bufler P., Dinarello C.A. (2010). IL-37 is a fundamental inhibitor of innate immunity. Nat. Immunol..

[32] Bulau A.M., Nold M.F., Li S., Nold-Petry C.A., Fink M., Mansell A., Schwerd T., Hong J., Rubartelli A., Dinarello C.A. (2014). Role of caspase-1 in nuclear translocation of IL-37, release of the cytokine, and IL-37 inhibition of innate immune responses. Proc. Natl. Acad. Sci. USA.

[33] Rudloff I., Cho S.X., Lao J.C., Ngo D., McKenzie M., Nold-Petry C.A., Nold M.F. (2017). Monocytes and dendritic cells are the primary sources of interleukin 37 in human immune cells. J. Leukoc. Biol..

[34] Nold-Petry C.A., Lo C.Y., Rudloff I., Elgass K.D., Li S., Gantier M.P., Lotz-Havla A.S., Gersting S.W., Cho S.X., Lao J.C. (2015). IL-37 requires the receptors IL-18Ralpha and IL-1R8 (SIGIRR) to carry out its multifaceted anti-inflammatory program upon innate signal transduction. Nat. Immunol..

[35] Shuai X., Wei-min L., Tong Y.L., Dong N., Sheng Z.Y., Yao Y.M. (2015). Expression of IL-37 contributes to the immunosuppressive property of human CD4+CD25+ regulatory T cells. Sci. Rep..

[36] Osborne D.G., Domenico J., Fujita M. (2022). Expression of IL-37 Induces a Regulatory T-Cell-like Phenotype and Function in Jurkat Cells. Cells.

[37] Wang Z., Zeng F.L., Hu Y.W., Wang X.Y., Zhao F.L., Zhou P., Hu J., Xiao Y.Y., Hu Z.L., Guo M.F. (2022). Interleukin-37 promotes colitis-associated carcinogenesis via SIGIRR-mediated cytotoxic T cells dysfunction. Signal Transduct. Target Ther..

[38] McNamee E.N., Masterson J.C., Jedlicka P., McManus M., Grenz A., Collins C.B., Nold M.F., Nold-Petry C., Bufler P., Dinarello C.A. (2011). Interleukin 37 expression protects mice from colitis. Proc. Natl. Acad. Sci. USA.

[39] Cho S.X., Rudloff I., Lao J.C., Pang M.A., Goldberg R., Bui C.B., McLean C.A., Stock M., Klassert T.E., Slevogt H. (2020). Characterization of the pathoimmunology of necrotizing enterocolitis reveals novel therapeutic opportunities. Nat. Commun..

[40] Chen Z., Wang S., Li L., Huang Z., Ma K. (2018). Anti-Inflammatory Effect of IL-37-Producing T-Cell Population in DSS-Induced Chronic Inflammatory Bowel Disease in Mice. Int. J. Mol. Sci..

[41] Cong J., Wu D., Dai H., Ma Y., Liao C., Li L., Ye L., Huang Z. (2022). Interleukin-37 exacerbates experimental colitis in an intestinal microbiome-dependent fashion. Theranostics.

[42] Li Y., Wang Y., Liu Y., Wang Y., Zuo X., Li Y., Lu X. (2014). The possible role of the novel cytokines il-35 and il-37 in inflammatory bowel disease. Mediat. Inflamm..

[43] Imaeda H., Takahashi K., Fujimoto T., Kasumi E., Ban H., Bamba S., Sonoda H., Shimizu T., Fujiyama Y., Andoh A. (2013). Epithelial expression of interleukin-37b in inflammatory bowel disease. Clin. Exp. Immunol..

[44] Wang W.Q., Dong K., Zhou L., Jiao G.H., Zhu C.Z., Li W.W., Yu G., Wu W.T., Chen S., Sun Z.N. (2015). IL-37b gene transfer enhances the therapeutic efficacy of mesenchumal stromal cells in DSS-induced colitis mice. Acta Pharmacol. Sin..

[45] Krohn L., Azabdaftari A., Heuberger J., Hudert C., Zilbauer M., Breiderhoff T., Bufler P. (2023). Modulation of intestinal IL-37 expression and its impact on the epithelial innate immune response and barrier integrity. Front. Immunol..

[46] Strauss L., Whiteside T.L., Knights A., Bergmann C., Knuth A., Zippelius A. (2007). Selective survival of naturally occurring human CD4+CD25+Foxp3+ regulatory T cells cultured with rapamycin. J. Immunol..

[47] Neurath M.F. (2014). Cytokines in inflammatory bowel disease. Nat. Rev. Immunol..

[48] Rudloff I., Godsell J., Nold-Petry C.A., Harris J., Hoi A., Morand E.F., Nold M.F. (2015). Brief Report: Interleukin-38 Exerts Antiinflammatory Functions and Is Associated With Disease Activity in Systemic Lupus Erythematosus. Arthritis Rheumatol..

[49] Bulek K., Swaidani S., Qin J., Lu Y., Gulen M.F., Herjan T., Min B., Kastelein R.A., Aronica M., Kosz-Vnenchak M. (2009). The essential role of single Ig IL-1 receptor-related molecule/Toll IL-1R8 in regulation of Th2 immune response. J. Immunol..

[50] Nold-Petry C.A., Nold M.F. (2022). Rationale for IL-37 as a novel therapeutic agent in inflammation. Expert Rev. Clin. Immunol..

[51] Fonseca-Camarillo G., Furuzawa-Carballeda J., Yamamoto-Furusho J.K. (2015). Interleukin 35 (IL-35) and IL-37: Intestinal and peripheral expression by T and B regulatory cells in patients with Inflammatory Bowel Disease. Cytokine.

[52] Seidelin J.B., Bjerrum J.T., Coskun M., Widjaya B., Vainer B., Nielsen O.H. (2010). IL-33 is upregulated in colonocytes of ulcerative colitis. Immunol. Lett..

[53] Ueno-Shuto K., Kato K., Tasaki Y., Sato M., Sato K., Uchida Y., Sakai H., Ono T., Suico M.A., Mitsutake K. (2014). Lipopolysaccharide decreases single immunoglobulin interleukin-1 receptor-related molecule (SIGIRR) expression by suppressing specificity protein 1 (Sp1) via the Toll-like receptor 4 (TLR4)-p38 pathway in monocytes and neutrophils. J. Biol. Chem..

[54] Li L., Wei J., Suber T.L., Ye Q., Miao J., Li S., Taleb S.J., Tran K.C., Tamaskar A.S., Zhao J. (2021). IL-37-induced activation of glycogen synthase kinase 3beta promotes IL-1R8/Sigirr phosphorylation, internalization, and degradation in lung epithelial cells. J. Cell Physiol..

[55] Seo H.H., Lee C.Y., Lee J., Lim S., Choi E., Park J.C., Lee S., Hwang K.C. (2016). The role of nuclear factor of activated T cells during phorbol myristate acetate-induced cardiac differentiation of mesenchymal stem cells. Stem. Cell Res. Ther..

[56] Gulen M.F., Kang Z., Bulek K., Youzhong W., Kim T.W., Chen Y., Altuntas C.Z., Sass Bak-Jensen K., McGeachy M.J., Do J.S. (2010). The receptor SIGIRR suppresses Th17 cell proliferation via inhibition of the interleukin-1 receptor pathway and mTOR kinase activation. Immunity.

[57] Sorkin A., Duex J.E. (2010). Quantitative analysis of endocytosis and turnover of epidermal growth factor (EGF) and EGF receptor. Curr. Protoc. Cell Biol..

[58] Li L., Wei J., Li S., Jacko A.M., Weathington N.M., Mallampalli R.K., Zhao J., Zhao Y. (2019). The deubiquitinase USP13 stabilizes the anti-inflammatory receptor IL-1R8/Sigirr to suppress lung inflammation. EBioMedicine.

[59] Huangfu W.C., Fuchs S.Y. (2010). Ubiquitination-dependent regulation of signaling receptors in cancer. Genes Cancer.

[60] Rajagopal S., Shenoy S.K. (2018). GPCR desensitization: Acute and prolonged phases. Cell Signal..

[61] Monaco G., Lee B., Xu W., Mustafah S., Hwang Y.Y., Carre C., Burdin N., Visan L., Ceccarelli M., Poidinger M. (2019). RNA-Seq Signatures Normalized by mRNA Abundance Allow Absolute Deconvolution of Human Immune Cell Types. Cell Rep..

[62] Fortino V., Wisgrill L., Werner P., Suomela S., Linder N., Jalonen E., Suomalainen A., Marwah V., Kero M., Pesonen M. (2020). Machine-learning-driven biomarker discovery for the discrimination between allergic and irritant contact dermatitis. Proc. Natl. Acad. Sci. USA.

[63] Nah J., Seong R.H. (2022). Kruppel-like factor 4 regulates the cytolytic effector function of exhausted CD8 T cells. Sci. Adv..

[64] Zhou L., Zhu L., Wu X., Hu S., Zhang S., Ning M., Yu J., Chen M. (2023). Decreased TMIGD1 aggravates colitis and intestinal barrier dysfunction via the BANF1-NF-kappaB pathway in Crohn’s disease. BMC Med..

[65] Vieujean S., Caron B., Haghnejad V., Jouzeau J.Y., Netter P., Heba A.C., Ndiaye N.C., Moulin D., Barreto G., Danese S. (2022). Impact of the Exposome on the Epigenome in Inflammatory Bowel Disease Patients and Animal Models. Int. J. Mol. Sci..

[66] Sun Y., Zhu X., Chen X., Liu H., Xu Y., Chu Y., Wang G., Liu X. (2014). The mediator subunit Med23 contributes to controlling T-cell activation and prevents autoimmunity. Nat. Commun..

[67] Levi B.Z., Hashmueli S., Gleit-Kielmanowicz M., Azriel A., Meraro D. (2002). ICSBP/IRF-8 transactivation: A tale of protein-protein interaction. J. Interferon. Cytokine Res..

[68] Donnelly R.P., Finlay D.K. (2015). Glucose, glycolysis and lymphocyte responses. Mol. Immunol..

[69] Lee H., Jeon J.H., Lee Y.J., Kim M.J., Kwon W.H., Chanda D., Thoudam T., Pagire H.S., Pagire S.H., Ahn J.H. (2023). Inhibition of Pyruvate Dehydrogenase Kinase 4 in CD4(+) T Cells Ameliorates Intestinal Inflammation. Cell Mol. Gastroenterol. Hepatol..

[70] Chen Y., Li X., Sun R., Ji J., Yang F., Tian W., Ji W., Huang Q. (2022). A broad cuproptosis landscape in inflammatory bowel disease. Front. Immunol..

[71] Yang C., Wang W., Li S., Qiao Z., Ma X., Yang M., Zhang J., Cao L., Yao S., Yang Z. (2023). Identification of cuproptosis hub genes contributing to the immune microenvironment in ulcerative colitis using bioinformatic analysis and experimental verification. Front. Immunol..

[72] Brenna O., Bruland T., Furnes M.W., Granlund A., Drozdov I., Emgard J., Bronstad G., Kidd M., Sandvik A.K., Gustafsson B.I. (2015). The guanylate cyclase-C signaling pathway is down-regulated in inflammatory bowel disease. Scand. J. Gastroenterol..

[73] Camilleri M. (2015). Guanylate cyclase C agonists: Emerging gastrointestinal therapies and actions. Gastroenterology.

[74] Desreumaux P., Foussat A., Allez M., Beaugerie L., Hebuterne X., Bouhnik Y., Nachury M., Brun V., Bastian H., Belmonte N. (2012). Safety and Efficacy of Antigen-Specific Regulatory T-Cell Therapy for Patients With Refractory Crohn’s Disease. Gastroenterology.

[75] Ellisdon A.M., Nold-Petry C.A., D’Andrea L., Cho S.X., Lao J.C., Rudloff I., Ngo D., Lo C.Y., Soares da Costa T.P., Perugini M.A. (2017). Homodimerization attenuates the anti-inflammatory activity of interleukin-37. Sci. Immunol..

[76] Tresoldi E., Dell’Albani I., Stabilini A., Jofra T., Valle A., Gagliani N., Bondanza A., Roncarolo M.G., Battaglia M. (2011). Stability of human rapamycin-expanded CD4+CD25+ T regulatory cells. Haematologica.

[77] Xu Y., Ou J., Zhang C., Chen J., Chen J., Li A., Huang B., Zhao X. (2024). Rapamycin promotes the intestinal barrier repair in ulcerative colitis via the mTOR/PBLD/AMOT signaling pathway. Biochim. Biophys. Acta Mol. Basis Dis..

[78] Irving P.M., Seah D., Centritto A., Clough J., Canavan J., Goldberg R., Prevost A.T., Vasconcelos J.C., Lyne M., Palmer Joyce J. (2024). P999 First in human treatment of Crohn’s disease with autologous ex-vivo expanded polyclonal, gut-targeted regulatory T cells: Initial results of the TRIBUTE feasibility study. J. Crohn’s Colitis.

[79] Galaxy C. (2024). The Galaxy platform for accessible, reproducible, and collaborative data analyses: 2024 update. Nucleic Acids Res..

[80] Andrews S. (2010). FastQC A Quality Control tool for High Throughput Sequence Data.

[81] Ewels P., Magnusson M., Lundin S., Kaller M. (2016). MultiQC: Summarize analysis results for multiple tools and samples in a single report. Bioinformatics.

[82] Martin M. (2011). Cutadapt removes adapter sequences from high-throughput sequencing reads. EMBnet J..

[83] Kim D., Langmead B., Salzberg S.L. (2015). HISAT: A fast spliced aligner with low memory requirements. Nat. Methods.

[84] Liao Y., Smyth G.K., Shi W. (2014). featureCounts: An efficient general purpose program for assigning sequence reads to genomic features. Bioinformatics.

[85] The R Core Team (2024). R: A Language and Environment for Statistical Computing.

[86] Love M.I., Huber W., Anders S. (2014). Moderated estimation of fold change and dispersion for RNA-seq data with DESeq2. Genome Biol..

[87] Blighe K., Rana S., Lewis M. (2018). EnhancedVolcano: Publication-Ready Volcano Plots with Enhanced Colouring and Labeling. https://bioconductor.org/packages/EnhancedVolcano.

