# Supplementary material for: Characteristics of T-Cells Expressing IL-37 and Its Receptors in Inflammatory Bowel Disease
Source: Int J Mol Sci. 2026 Feb 4;27(3):1540. doi: 10.3390/ijms27031540 (PMC12898301; doi:10.3390/ijms27031540)
Supplement: Supplementary file 1 [file ijms-27-01540-s001.zip › ijms-4077037-supplementary.pdf]

Characteristics of T-Cells Expressing IL-37 and Its Receptors in Inflammatory Bowel Disease

Indiana Zorkau [1,†], Peter J. Eggenhuizen [1,*,†], Marie Lee [1], Steven X. Cho [2,3], Kylie R. James [4,5], Andrew M. Ellisdon [6], James C. Whisstock [6], Joshua D. Ooi [1], Marcel F. Nold [2,3,7,‡], Claudia A. Nold-Petry [2,3,‡] and Rimma Goldberg [1,8,*,‡]

1 Centre for Inflammatory Diseases, Department of Medicine, School of Clinical Sciences, Monash University, Clayton, VIC 3168, Australia; indiana.zorkau@monash.edu (I.Z.); marie.lee@monash.edu (M.L.); joshua.ooi@monash.edu (J.D.O.)
2 The Ritchie Centre, Hudson Institute of Medical Research, Clayton, VIC 3168, Australia; steven.cho1@hudson.org.au (S.X.C.); marcel.nold@monash.edu (M.F.N.); claudia.nold@hudson.org.au (C.A.N.-P.)
3 Department of Paediatrics, Monash University, Clayton, VIC 3168, Australia
4 Garvan Institute of Medical Research, Darlinghurst, NSW 2010, Australia; k.james@garvan.org.au (K.R.J)
5 School of Biomedical Sciences, University of New South Wales, Sydney, NSW 2052, Australia
6 Biomedicine Discovery Institute, Monash University, Clayton, VIC 3800, Australia; andrew.ellisdon@monash.edu (A.M.E.); james.whisstock@monash.edu (J.C.W.)
7 Monash Children's Hospital, Clayton, VIC 3168, Australia
8 Department of Gastroenterology, Monash Health, Clayton, VIC 3168, Australia
* Correspondence: peter.eggenhuizen@monash.edu (P.J.E.); rimma.goldberg@monash.edu (R.G.)
† These authors contributed equally to this work.
‡ These authors jointly supervised this work.

Supplementary Data

Contents:

Supplementary Figures:

Supplementary Figure S1. Representative gating strategy of T-cells. Flow cytometry representative dot plots showing

Supplementary Figure S2. Percentage of $CD4^+$ T-cells and $CD4^+$ T-cell subsets from PBMC

Supplementary Figure S3. IL-37 expression in each disease state is increased in Treg cells compared to Th1 cells.

Supplementary Figure S4. Co-expression of IL-1R5 and IL-1R8 in each disease state is increased in Treg cells compared to $CD8^+$ T-cells.

Supplementary Figure S5. Principal component analysis of gene expression across all groups.

Supplementary Figure S6. Representative gating strategy of ex-vivo assays.

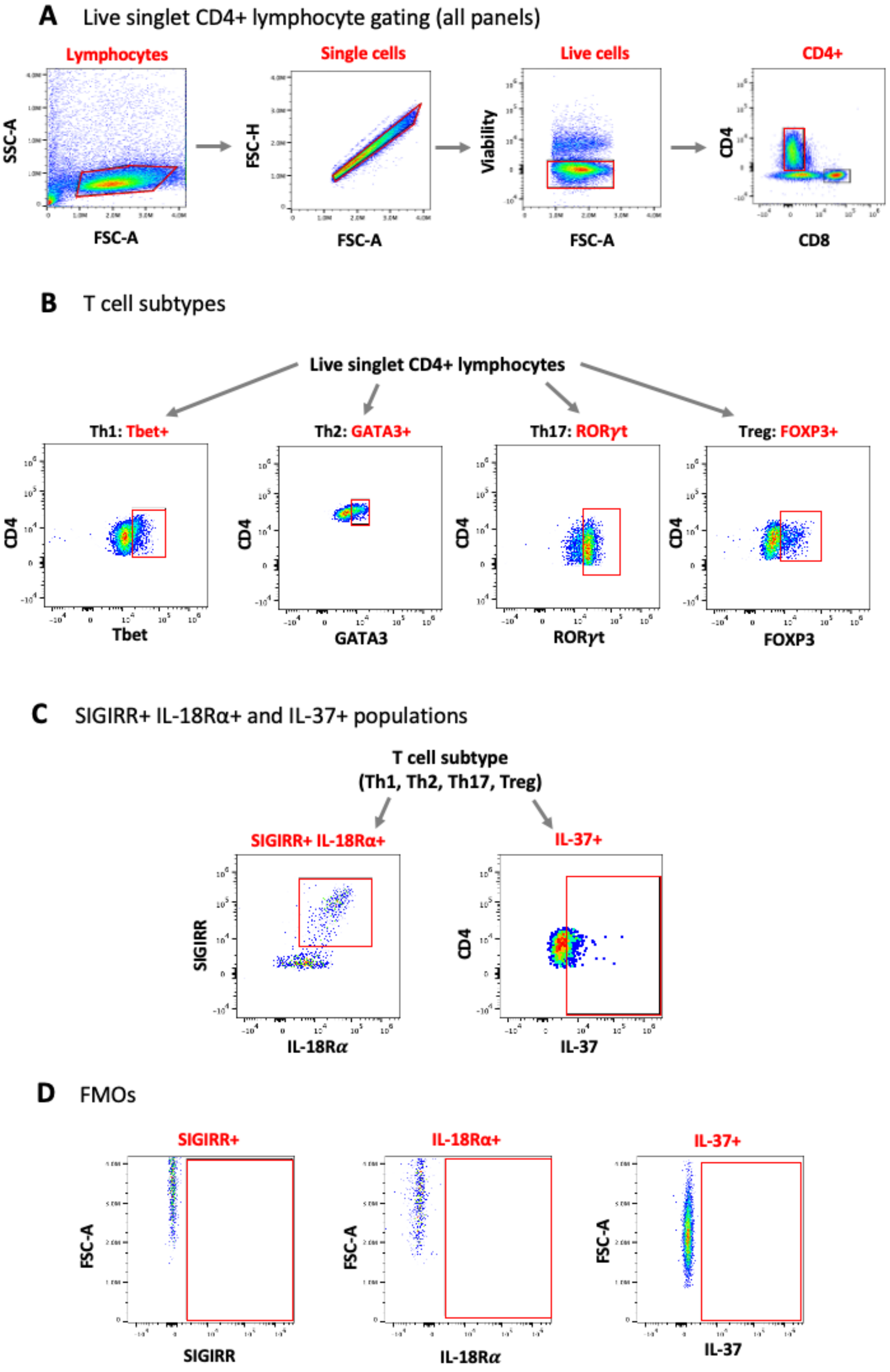

Supplementary Figure S1. Representative gating strategy of T-cells. Flow cytometry representative dot plots showing (A) Gating of CD4^{+} and CD8^{+} T-cells, (B) gating of CD4^{+} T-cell subtypes, (C) gating of IL-1R5^{+}IL-1R8^{+} (IL-18Rα^{+}SIGIRR^{+}) and IL-37^{+} populations from the CD4^{+} T-cell subtypes and (D) FMOs of IL-1R8 (SIGIRR), IL-1R5 (IL-18Rα) and IL-37.

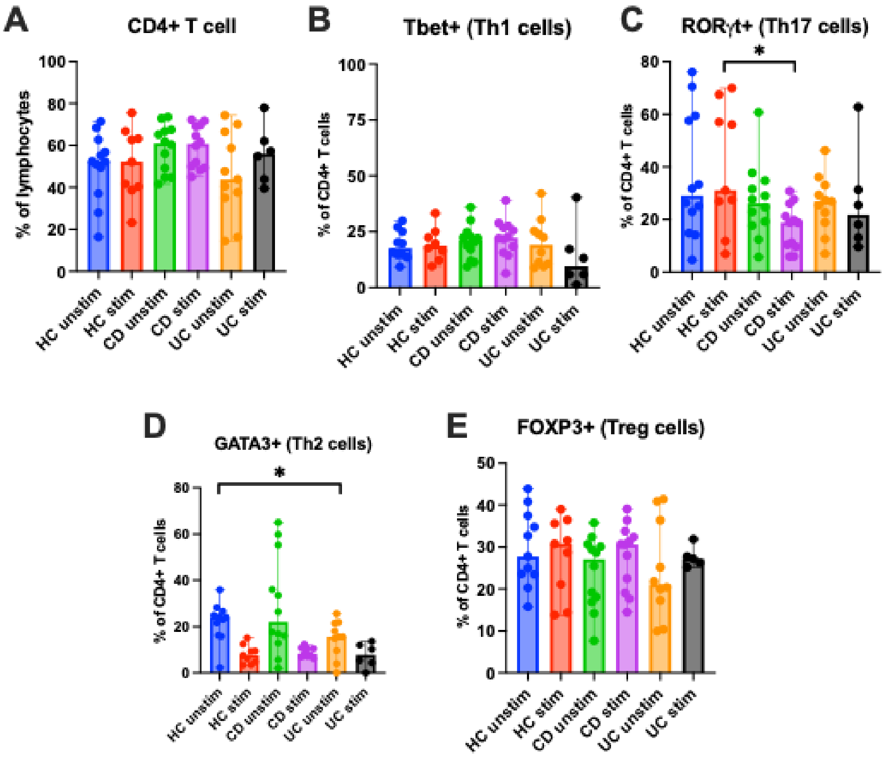

Supplementary Figure S2. Percentage of CD4$^+$ T-cells and CD4$^+$ T-cell subsets from PBMC

PBMCs were either unstimulated (unstim) or stimulated (stim) with PMA (50 ng/mL) and Ionomycin (750 ng/mL), with the addition of brefeldin A (10 µg/mL) for 4 hours. Percentage of (A) CD4$^+$ T-cells of total lymphocytes, (B) Tbet$^+$ Th1 cells of CD4$^+$ T-cells, (C) RORγt$^+$ Th17 cells of CD4$^+$ T-cells), (D) GATA3$^+$ Th2 cells of CD4$^+$ T-cells) and (E) FOXP3$^+$ Treg cells of CD4$^+$ T-cells. Mann-Whitney U test was used to determine statistical significance among patient groups. Healthy control (HC) n = 12, Crohn's disease (CD) n = 12, ulcerative colitis (UC) n = 11. Outliers were removed using the ROUT outliers test. Data is represented as median ± range. * $p < 0.05$.

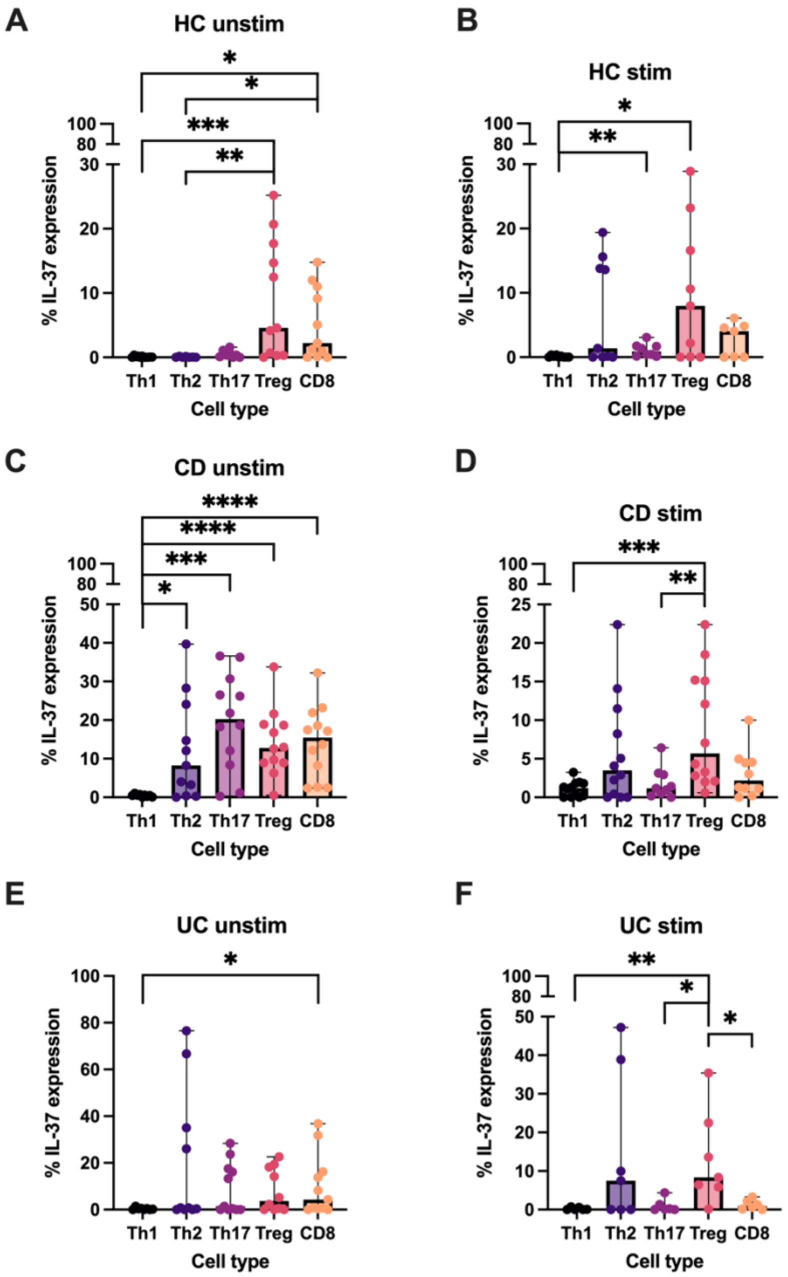

Supplementary Figure S3. IL-37 expression in each disease state is increased in Treg cells compared to Th1 cells. PBMCs were either left untreated or stimulated with PMA (50 ng/mL) and Ionomycin (750 ng/mL), with the addition of brefeldin A (10 µg/mL) for 4 hours. Expression of IL-37 in each cell type in (A) HC unstimulated cells, (B) HC stimulated cells, (C) CD unstimulated cells, (D) CD stimulated cells, (E) UC unstimulated cells, (F) UC stimulated cells. Mann-Whitney U test was used to determine statistical significance among patient groups. n = 12 HC, n = 12 CD, n = 11 UC. Outliers were removed using the ROUT outliers test. Data is represented as median ± range. * $p < 0.05$, ** $p < 0.005$, *** $p < 0.0005$, **** $p < 0.0001$.

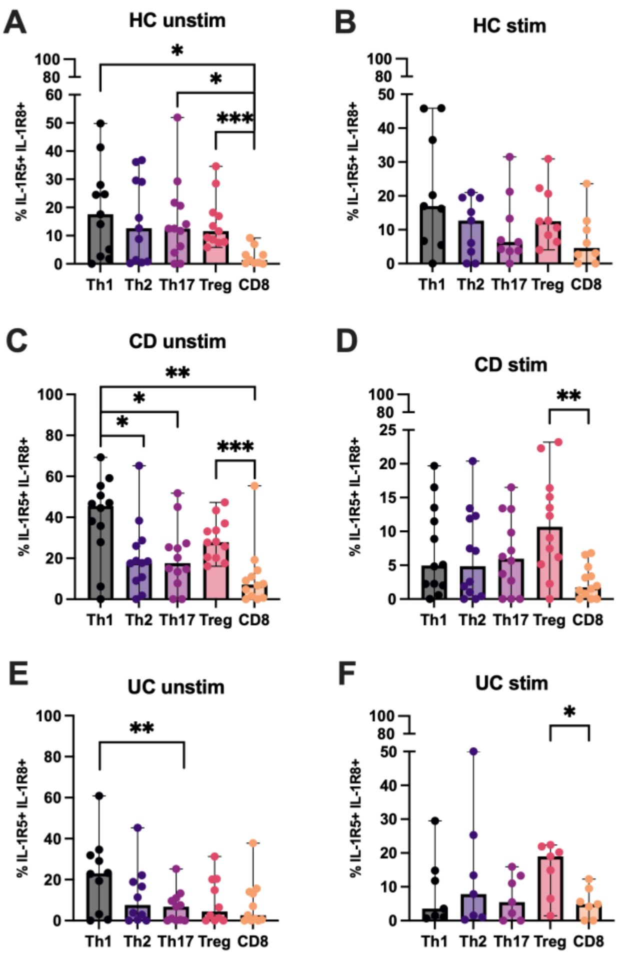

Supplementary Figure S4. Co-expression of IL-1R5 and IL-1R8 in each disease state is increased in Treg cells compared to CD8^{+} T-cells. PBMCs were either left unstimulated or stimulated with PMA (50 ng/mL) and Ionomycin (750 ng/mL) with brefeldin A (10 µg/mL) for 4 hours. Co-expression of IL-1R5 and IL-1R8 in each cell type in (A) HC unstimulated cells, (B) HC stimulated cells, (C) CD unstimulated cells, (D) CD stimulated cells, (E) UC unstimulated cells. (F) UC stimulated cells. Mann-Whitney U test was used to determine statistical significance among patient groups. n = 12 HC, n = 12 CD, n = 11 UC. Outliers were removed using the ROUT outliers test. Data is represented as median ± range. * $p < .05$, ** $p < 0.005$, *** $p < 0.0005$.

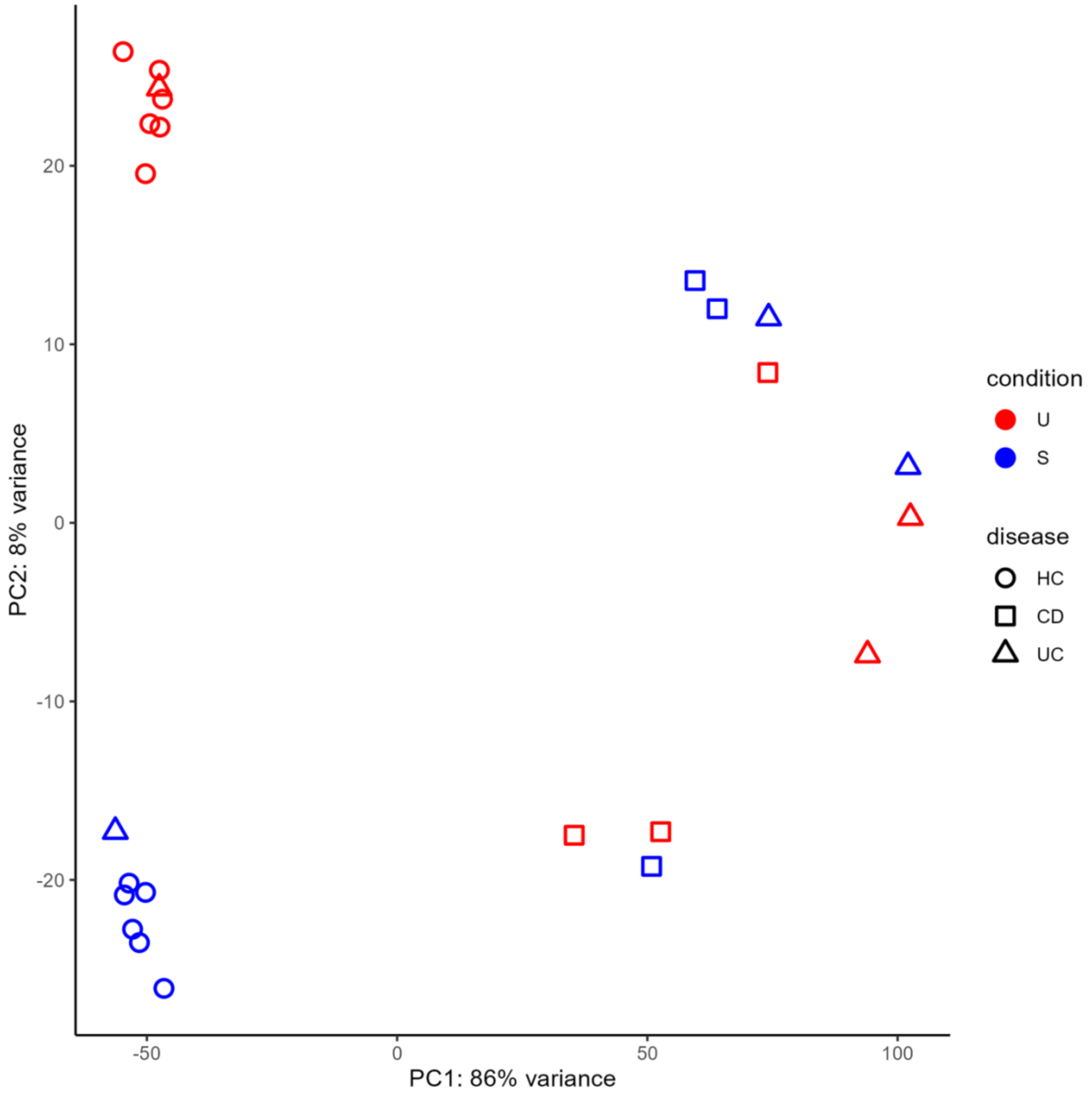

Supplementary Figure S5. Principal component analysis of gene expression across all groups. Each point represents one sample. Point shape indicates disease (HC, healthy control; CD, Crohn's disease; UC, ulcerative colitis), with colour indicating stimulated (S, blue) or unstimulated (U, red) samples. The percentage of variance explained by each principal component is shown on the axes.

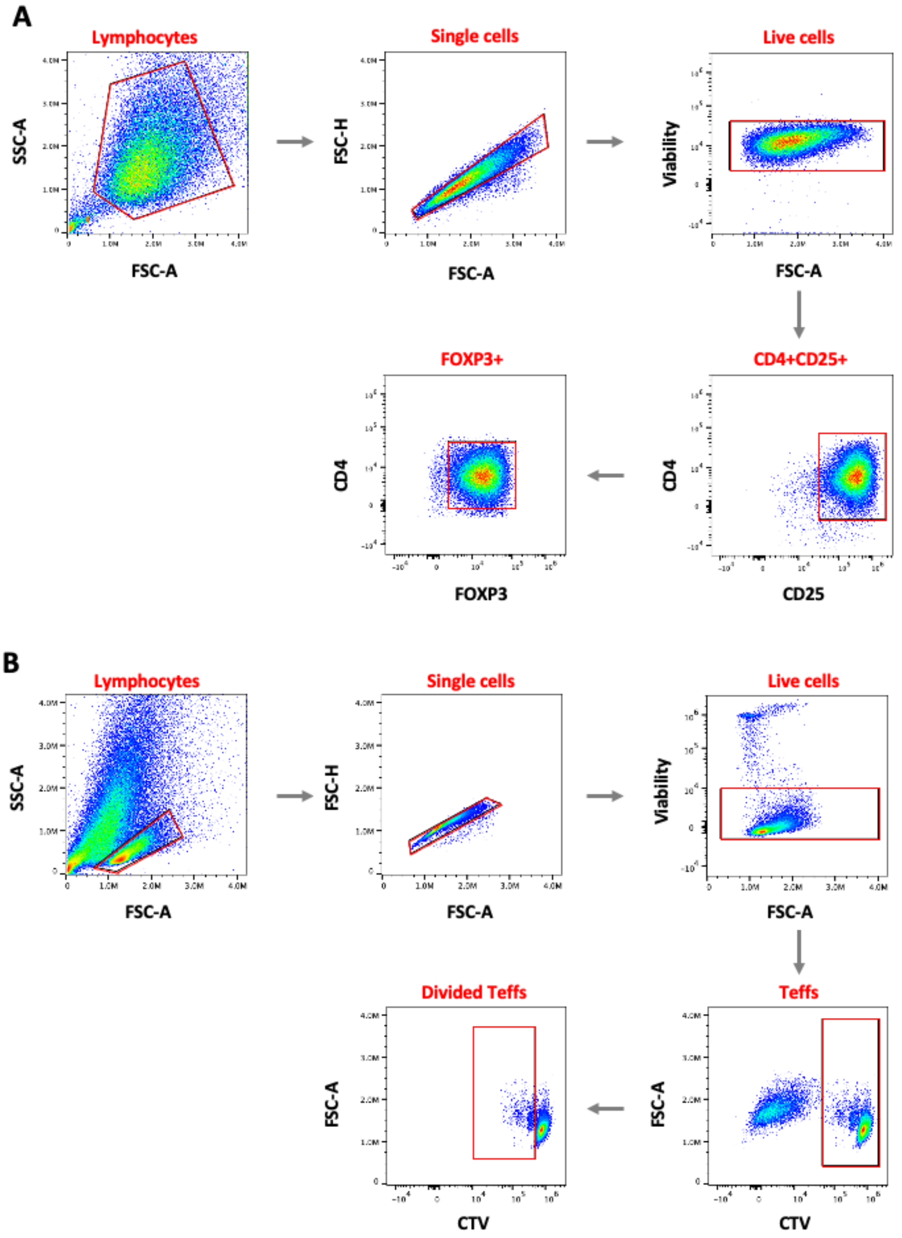

Supplementary Figure S6. Representative gating strategy of ex-vivo assays. (A) Representative flow cytometry dot plots of the gating strategy for the assessment of Treg phenotypic stability. (B) Representative flow cytometry dot plots of the gating strategy of the division of T effector cells (Teffs) identified by staining of proliferation dye CellTrace Violet (CTV).
